# Supplementary material for: Conversion to secondary progressive multiple sclerosis: Multistakeholder experiences and needs in Italy
Source: PLoS One. 2020 Feb 13;15(2):e0228587. doi: 10.1371/journal.pone.0228587 (PMC7018010; doi:10.1371/journal.pone.0228587)
Supplement: S1 Appendix — (PDF) [file pone.0228587.s001.pdf]

S1 Appendix - COREQ checklist

Consolidated criteria for reporting qualitative studies (COREQ): 32-item checklist

Developed from:
Tong A, Sainsbury P, Craig J. Consolidated criteria for reporting qualitative research (COREQ): a 32-item checklist for interviews and focus groups. *International Journal for Quality in Health Care*. 2007. Volume 19, Number 6: pp. 349 – 357

No. Item	Guide questions/description	Reported on Page #
**Domain 1: Research team and reflexivity**		
Personal Characteristics		
1. Interviewer/facilitator	Which author/s conducted the inter view or focus group?	PSIs. **Methods/Interviews, p. 4**: "The interviews were conducted by a psychologist experienced in qualitative research at the MS Center of Milan (AMG), Chieti and Bari (EP)".<br><br>FGMs. **Methods/Focus group meetings, p. 4**: "Each FGM was planned to have 6 to 10 participants plus two moderators (AMG and EP) and run at the Fondazione IRCCS Istituto Neurologico C. Besta"
2. Credentials	What were the researcher's credentials? E.g. PhD, MD	Ambra Mara Giovannetti: PsyD, Erika Pietrolongo: PhD and PsyD.
3. Occupation	What was their occupation at the time of the study?	Ambra Mara Giovannetti: psychologist, researcher at the Fondazione IRCCS Istituto Neurologico C. Besta; Erika Pietrolongo: psychologist researcher at G d'Annunzio University of Chieti-Pescara.
4. Gender	Was the researcher male or female?	They are both female.
5. Experience and training	What experience or training did the researcher have?	They have previously participated in qualitative study running PSIs and FGMs and performing data analysis. In addition, a dedicated training was run for the ManTra project. Details are presented in **Appendix 3 – Audit trail.**
Relationship with		

participants		
6. Relationship established	Was a relationship established prior to study commencement?	Both interviewers and facilitators were not acquainted to the interviewers prior to the interview. They both have already meet some HPs or neurologist prior to the FGMs conduction.
7. Participant knowledge of the interviewer	What did the participants know about the researcher? e.g. personal goals, reasons for doing the research	**Methods p. 4**: "Before PSIs/FGMs began, participants were informed of study aims and requirement and signed the written consent, in accordance with the Helsinki Declaration and EU Good Clinical Practice guidelines."<br><br>**Methods/Interviews, p. 4**: "The interviewer presented the purpose of the PSI and then posed the questions. Patients were encouraged to provide the interviewer with their opinion and perspective on the transition to SPMS and their needs."<br><br>**Methods/Focus group meetings, p. 5**: "The principal moderator first explained the aim of the meeting, asked participants to introduce themselves. She then introduced each topic in turn, facilitating the discussion. After all pre-specified topics had been discussed, the moderator summarized the key points, and asked for further comments".
8. Interviewer characteristics	What characteristics were reported about the inter viewer/facilitator? e.g. Bias, assumptions, reasons and interests in the research topic	Interviewers and facilitators were member of the Qualitative Analysis Panel. EP was specifically dedicated to interviews and focus group and she did not participate to any other study activities. They were not part of the Steering Committee.<br>AMG was study CO-PI.

**Domain 2: study design**		
Theoretical framework		
9. Methodological orientation and Theory	What methodological orientation was stated to underpin the study? e.g. grounded theory, discourse analysis, ethnography, phenomenology, content analysis	**Methods/Analysis, p. 5**: "The methods of framework analysis […] the Psychologist Working Group.
Participant selection		PSIs. **Methods/Interviews, p. 4**<br><br>FGMs. **Methods/Focus group meetings, p. 5**
10. Sampling	How were participants selected? e.g. purposive, convenience, consecutive, snowball	**Methods, p. 4**: "Participants'enrollment followed a purposive sampling technique. To cover a range of experiences, they were selected from the three geographic areas of Italy (North, Central, South), and varied in terms of gender, education and (for patients) disease severity (EDSS score) [Kurtzke 1983]."
11. Method of approach	How were participants approached? e.g. face-to-face, telephone, mail, email	**Methods, p. 4**: "Patients (and SOs) were contacted by their neurologist and who showed interest in participating in the study were then contacted by the interviewer. Health care professionals were invited by the study coordinator."
12. Sample size	How many participants were in the study?	**Results/ Participants and setting, p. 5-6 (and Table 1)**
13. Non-participation	How many people refused to participate or dropped out? Reasons?	Three SOs refused to participate: two because

		of work commitment (North); one because of the geographical distance (Central).
Setting		
14. Setting of data collection	Where was the data collected? e.g. home, clinic, workplace	PSIs.<br>**Methods/Interviews, p. 4**: "The interviews were conducted by a psychologist experienced in qualitative research at the MS Center of Milan (AMG), Chieti and Bari (EP)".<br><br>FGMs.<br>**Methods/Focus group meetings, p. 4**: "Each FGM was planned to have 6 to 10 participants plus two moderators (AMG and EP) and run at the Fondazione IRCCS Istituto Neurologico C. Besta"
15. Presence of non-participants	Was anyone else present besides the participants and researchers?	**No**
16. Description of sample	What are the important characteristics of the sample? e.g. demographic data, date	**Results/ Participants and setting, p. 5-6 (and Table 1)**
Data collection		
17. Interview guide	Were questions, prompts, guides provided by the authors? Was it pilot tested?	**Methods, p. 4**: "AG, AMG, EP and CB devised the PSI and FGM guides (Appendix 2) with input from the Psychologist Working Group, five MS psychologists from various institutions, who participated in a four-day training course in qualitative research methods where the ManTra project was the "case study". No

		changes were needed in PSI and FGMs guides, after piloting them". More details are provided in **Appendix 2.**
18. Repeat interviews	Were repeat inter views carried out? If yes, how many?	
19. Audio/visual recording	Did the research use audio or visual recording to collect the data?	**Methods, p. 4**: "PSIs and FGMs were audiorecorded and verbatim transcribed. A complete audit trail of the study is reported in Appendix 3."
20. Field notes	Were field notes made during and/or after the inter view or focus group?	**Methods/ Focus group meetings, p. 5**: "The principal moderator first explained the aim of the meeting, asked participants to introduce themselves. She then introduced each topic in turn, facilitating the discussion. After all pre-specified topics had been discussed, the moderator summarized the key points, and asked for further comments. The co-moderator took notes and oversaw the audio recording. By two weeks from the FGMs, participants received a report of the meeting for review (respondent validation)."
21. Duration	What was the duration of the inter views or focus group?	**Results/ Participants and setting, p. 5**: "Between March and August 2017, 15 interviews (mean duration 51.1 minutes; range 30-67) were conducted at the three participating MS centers. All the three

		FGMs took place between May and September 2017 at the Fondazione IRCCS Istituto Neurologico C. Besta (SOs, 107 minutes; HPs, 108 minutes; N, 109 minutes).”
22. Data saturation	Was data saturation discussed?	**Methods/Analysis p. 5**: “Once this process was completed, AMG and EP presented the results of the analysis that were discussed with the Qualitative Analysis Panel and the Psychologist Working Group”.
23. Transcripts returned	Were transcripts returned to participants for comment and/or correction?	Yes, for FGMs. **Methods/Focus group meetings, p. 5**: “By two weeks from the FGMs, participants received a report of the meeting for review (respondent validation).”
**Domain 3: analysis and findings**		
Data analysis		
24. Number of data coders	How many data coders coded the data?	Two (AMG and EP). **Methods/Analysis, p. 5**: “Once this process was completed, AMG and EP presented the results of the analysis that were discussed with the Qualitative Analysis Panel and the Psychologist Working Group.”
25. Description of the coding tree	Did authors provide a description of the coding tree?	**Methods/Analysis, p. 5**: “The transcripts were analysed in six successive steps (see below), each of which embodies an increasing level of generalization

		[Mc Cracken 1988]. To enhance the validity of this process, two researchers analysed the transcripts independently (FGMs, steps 1–4; PSIs, steps1–5) and jointly (step 6). Steps in the analysis:<br>1. The researcher identifies all propositions considered significant, without considering their relation to other parts of the transcript and appends comments to these significant propositions.<br>2. Comments are expanded and contextualised along with the entire PSI/FGM.<br>3. Relations between comments are established by reordering and regrouping them by subject.<br>4. Themes are extrapolated and hierarchically ordered into categories, moving from general concepts to more specific ones.<br>5. Each PSI/FGM transcript analysis is compared with the others to identify differences and commonalities in the themes<br>6. The analyses produced by the two researchers are compared, and a consensus is arrived at. Once this process was completed, AMG and

		EP presented the results of the analysis that were discussed with the Qualitative Analysis Panel and the Psychologist Working Group.”
26. Derivation of themes	Were themes identified in advance or derived from the data?	Derived from the data. **Methods/Analysis, p. 5**: “Framework analysis uses an inductive approach to identify, extract and analyse core themes [Denzin 2000; Crabtree 1992; Silverman 1993].”
27. Software	What software, if applicable, was used to manage the data?	We did not use any software.
28. Participant checking	Did participants provide feedback on the findings?	No.
Reporting		
29. Quotations presented	Were participant quotations presented to illustrate the themes/findings? Was each quotation identified? e.g. participant number	Yes. **Results/Qualitative findings, p. 7** & **Appendix 3 – Audit trail**. “The findings are presented by theme, with only the most relevant quotes to illustrate their derivation. The provenance of quotes is indicated as patient (P), SO, neurologist (N) or HP, with other information included as appropriate (sex, age, EDSS score [Kurtzke 1983], relation to patient, profession, and center). The complete list of quotes for each sub-category is reported in Appendix 3.”
30. Data and findings consistent	Was there consistency between the data presented and the findings?	**Yes.**

31. Clarity of major themes	Were major themes clearly presented in the findings?	Yes, we focused particularly on two of them: awareness of the transition and needs.
32. Clarity of minor themes	Is there a description of diverse cases or discussion of minor themes?	Yes. The **Appendix 3 – Audit trail** includes a description of each subcategories.

References

Crabtree BF, Miller WL. Doing qualitative research. London, UK: Sage Publications; 1992.

Denzin NK, Lincoln YS. Handbook of qualitative research. London, UK: Sage Publications; 2000.

Kurtzke JF. Rating neurologic impairment in multiple sclerosis: an expanded disability status scale (EDSS). Neurology 1983;33: 1444–52.

Mc Cracken G. The long interview. London, UK: Sage Publications; 1988.

Silverman D. Interpreting qualitative data. London, UK: Sage Publications; 1993.
