# Supplementary material for: Conversion to secondary progressive multiple sclerosis: Multistakeholder experiences and needs in Italy
Source: PLoS One. 2020 Feb 13;15(2):e0228587. doi: 10.1371/journal.pone.0228587 (PMC7018010; doi:10.1371/journal.pone.0228587)
Supplement: S2 Appendix — (PDF) [file pone.0228587.s002.pdf]

S2 Appendix – PSI and FGM guides

PSI guide

Impact of the reclassification

- **Could you please tell me about your experience with the transition from a relapsing remitting (RR) phase to a secondary progressive (SP) one?**
    - Have you perceived/Did you perceive any differences/changes?
    - When and how did you understand that your disease (phase) was changing/changed (transition from a RR form to a SP one)?
    - What have you made thinking that this change was going on/happened?
    - Did you expect this change?
    - Did you understand the transition was happening by yourself (before someone else told you)

- **Could you please tell me how was when you received the communication (or when you realized the transition happened) about the transition to a secondary progressive form?**
    - How was your experience? How did you feel?

- **What has been/was the impact of the reclassification?**
    - How was the impact on your daily life? What about your roles?
    - What about your family?

Management of the changes

- **Have you perceived any positive or negative changes since you received the reclassification to a SPMS?**

- **How have you dealt with the transition from a RR form to a SP one?**
    - Have you encountered any difficulties in dealing with this change?
    - Which kind of resources have you found (think about both your inner and the environmental resources)
    - Which has been the hardest time in this experience? How did you deal with that?
    - There have been any positive moments during this experience?

- **Have you noticed any changes in the quality and way of caring you received at the MS centre? Has your relationship with the centre changed? If yes, how has it changed?**
    - For example, did the frequency of the follow up visits change? Did you meet other health care professional (i.e. not only the neurologist, but also the physician, the urologist, the psychologist, etc.)?

Patients' strategies and needs

Please, think about the entire transition period you had or have lived. I mean the transition from a RR phase to SP one. In particular, think about: 1) when you have begun to perceive that something was

changing in the disease form (if you were aware of that); 2) when you spoke about the transition with an health care professional; 3) how you have dealt with this change in your daily life until now.

- **Have you found something useful to deal with the transition? If yes, what have you found useful?**
  - Please, think about different areas: information and communication; psychological support; management; social area, others.
  - Some hypotheses about what could be useful have been presented in literature, such as: informative materials; psychological support; self-help groups; interventions aimed at supporting patients' significant others; individual interviews/visits with some healthcare specialists. Do you think they could be of any help for?

- **Do you think that something could have helped you, but you did not received that? If yes, what are you thinking about? How do you think it could have helped you?**
  - Please, think about the following different areas: informative materials; psychological support; self-help groups; interventions aimed at supporting patients' significant others; individual interviews/visits with some healthcare specialists. Do you think they could be of any help?
  - Some hypotheses about what could be useful have been presented in literature, such as: informative materials; psychological support; self-help groups; interventions aimed at supporting patients' significant others; individual interviews/visits with some healthcare specialists. Do you think they could be of any help?
  - To who should be addressed the resource (i.e. the patient, the relevant others, the health care professional)?

- **What would you suggest to someone who is dealing with the transition to a SP phase?**

- **What would you suggest to the health care professional who is caring for someone who is dealing with the transition to a SP phase?**

- **Do you want to add something? Is there something I did not ask you and that you think it could be important to say?**

Conclusion

Provide the participant with a feedback about his/her self-efficacy and coping strategies used.
Thank the participant for the time She/He dedicated.

Neurologist FGM guide

Please, introduce yourself by telling us your name, profession and expertise and institution.

- **Could you please tell us about your experience with patients who have lived the transition to SPMS?**
    - How do you face this topic with the patients?
- **In your opinion, what are the needs of the MS patients living the transition to SPMS?**
- **Which difficulties/barriers have you encountered or could you encounter in satisfying patients needs during the transition?**
- **In your experience, what could help the patients in dealing better with the transition to SPMS?**
- **Have you (or your centre) activated dedicated resources for these patients?**
- **In your opinion, which kind of resource should we think about/build up for these patients?**
- **Is there anything you want to add?**

Other Health Care Professionals FGM guide

Please, introduce yourself by telling us your name, profession, expertise, institution and what kind of «contact» do you usually have with patients who live the transition

- **Could you please tell us about your experience with patients who have lived the transition to SPMS?**
- **In your opinion, what are the needs of the MS patients living the transition to SPMS?**
- **Which difficulties/barriers have you encountered or could you encounter in satisfying patients' needs during the transition?**
- **In your experience, what could help the patients in dealing better with the transition to SPMS?**
- **Have you (or your centre) activated dedicated resources for these patients?**
- **In your opinion, which kind of resource should we think about/build up for these patients?**
- **Is there anything you want to add?**

Significant Others FGM guide

Please, introduce yourself by telling us your name and your relationship with the person with SPMS.

- **How was your relative transition to SPMS?**
  - How did you discover the transition was happening? How did you recognise the transition was going on?
- **How was the living experience of your relative during the transition?**
- **How was the relation with the MS Centre during the transition to secondary progressive?**
  - Did you notice any differences? Did it change in any way?
- **What kind of difficulties have your relative had to deal with during the transition to SPMS?**
  - Which were his/her needs during the transition?
  - Which needs have been satisfied, which not?
- **Did something help your relative to deal with this change?**
  - Please, think about both personal strategies and external/environmental factors that may have played a relevant role.
- **What kind of resources/interventions could be useful for these needs?**
- **What would have helped you to support your relative in this transition phase?**
- **Is there anything you want to add?**
