# Supplementary material for: Conversion to secondary progressive multiple sclerosis: Multistakeholder experiences and needs in Italy
Source: PLoS One. 2020 Feb 13;15(2):e0228587. doi: 10.1371/journal.pone.0228587 (PMC7018010; doi:10.1371/journal.pone.0228587)
Supplement: S3 Appendix — (PDF) [file pone.0228587.s003.pdf]

S3 Appendix – Audit trail

GLOSSARY

AISM Italian Multiple Sclerosis Society
FGM focus group meeting
HP other health care professional
I interviewer
MS multiple sclerosis
N neurologist
P patient
PSI personal semistructured interview
SO significant other
SPMS secondary progressive multiple sclerosis

PSI AND FGM GUIDES DEVELOPMENT

AG, AMG, EP and CB devised the PSI and FGM guides with input from the Psychologist Working group during a four-day training programme held between February and September 2017 in Milan, Italy (25 CME credits). Ten psychologists from 6 Institutions (AISM Milano; IRCCS Besta; IRCCS San Raffaele; University of Bergamo; University of Chieti-Pescara; University of Milano) participated. The seminar covered the following:

- Day 1. ManTra study overview; introduction to qualitative research methods; results of the scoping review; principles of interview guide development; peer discussion; construction of PSI guides.
- Day 2. Review of PSI guides; introduction to holding FGMs; principles of FGM guide development; peer discussion; construction of FGM guides for neurologists and HPs.
- Day 3. Review of FGM guides for neurologists and HPs; construction of FGM guides for patient SOs; peer discussion; review of the FGM guide for patient SOs.
- Day 4. Qualitative data analysis.

The PSI and FGM guides are reported in Appendix 1.

DATA ANALYSIS

AMG and EP performed qualitative data analysis. The methods of framework analysis was applied to the PSI and FGM transcripts. Framework analysis uses an inductive approach to identify, extract and analyse core themes [1-3]. The transcripts were analysed in six successive steps (see below), each of which embodies an increasing level of generalization [4]. To enhance the validity of this process, two researchers analysed the transcripts independently (FGMs, steps 1–4; PSIs, steps 1–5) and jointly (step 6).
Steps in the analysis:

1. The researcher identifies all propositions considered significant, without considering their relation to other parts of the transcript and appends comments to these significant propositions.
2. Comments are expanded and contextualised along with the entire PSI/FGM.
3. Relations between comments are established by reordering and regrouping them by subject.
4. Themes are extrapolated and hierarchically ordered into categories, moving from general concepts to more specific ones.
5. Each PSI transcript analysis is compared with the others to identify common themes and also differences.
6. The analyses produced by the two researchers are compared, and a consensus is arrived at.

Once this process was completed, AMG and EP presented the results of the analysis, which were discussed with the Qualitative Analysis Panel and the Psychologist Working Group.

References

1. Denzin NK, Lincoln YS, eds. Handbook of qualitative research. London, UK: Sage, 2000.
2. Crabtree BF, Miller WL. Doing qualitative research. London, UK: Sage Publications, 1992.
3. Silverman D. Interpreting qualitative data. London, UK: Sage Publications, 1993.
4. Mc Cracken G. The long interview. London, UK: Sage Publications, 1988.

<u>CHARACTERISTICS OF THE PARTICIPANTS</u>

Patients

ID	Area of Italy	Sex	Age (years)	Education	Occupation	Marital status	Age at MS diagnosis (years)	Age at SP diagnosis (years)	EDSS score	Transition awareness
P01	North	M	46	Degree	Employed	Married	13	42	6.5	Yes
P02	North	F	60	Sec. sch.	Retired (age)	Married	44	57	4	No
P03	North	M	52	Sec. sch.	Employed	Separated/divorc.	23	50	6	Yes
P04	North	M	44	Degree	Employed	Married	35	43	6	Yes
P05	North	F	44	Degree	Employed	Married	18	43	6	No
P06	North	F	39	Degree	Employed	Married	26	38	7	Yes
P07	North	M	49	High sch.	Employed	Married	39	48	6	No
P08	Centre	M	52	Sec. sch.	Employed	Married	52	50	6	No
P09	Centre	F	60	High sch.	Retir. (disab.)	Married	58	58	6	No
P10	Centre	F	41	Degree	Employed	Married	40	40	4.5	Yes
P11	Centre	M	47	High sch.	Employed	Married	27	46,5	6.5	Yes
P12	South	F	56	Degree	Employed	Married	49	52	6.5	No
P13	South	F	42	Degree	Employed	Married	31	37	6.5	Yes
P14	South	F	59	High sch.	Retir. (disab.)	Married	50	56	5.5	No
P15	South	M	39	High sch.	Employed	Single	31	38	7	Yes

Patient significant others

ID	Area of Italy	Sex	Age (years)	Education	Occupation	Relationto patient	Working activity reduced	Assistance provided
SO01	North	F	48	Degree	Housewife	Partner	Completely	Part of the day
SO02	North	M	41	Degree	Employed	Partner	Not at all	Part of the day
SO03	North	F	42	High School	Housewife	Partner	Partially	Part of the day
SO04	North	F	49	PhD	Employed	Partner	Partially	Part of day/night
SO05	North	F	51	Degree	Unemployed	Partner	Completely	Part of the day
SO06	North	F	27	Degree	Employed	Daughter	Not at all	Part of the day
SO07	South	M	46	PhD	Employed	Partner	Not at all	All day long

Neurologists

ID	Area of Italy	Sex	Age (years)	MS expertise (years)	MS patients followed (previous 3 months)	SPMS patients followed (previous 3 months)
N01	Centre	F	49	20	90	10
N02	Centre	F	50	20	90	20
N03	South	F	50	25	200	20
N04	South	F	32	7	90	20
N05	North	F	34	3	60	5
N06	Centre	F	49	20	90	10
N07	Centre	F	50	20	90	20

Other health care professionals

ID	Area of Italy	Sex	Age (years)	Profession	MS expertise (years)	MS patients followed (previous 3 months)	SPMS patients followed (previous 3 months)
HP01	North	F	47	Psychologist	17	26	18
HP02	North	F	58	Nurse	30	70	70
HP03	North	F	56	Physiotherapist	15	50	15
HP04	North	F	48	Nurse	13	250	20
HP05	North	F	50	Social worker	12	50	20
HP06	North	M	46	Psychologist	15	6	Not applicable
HP07	Centre	F	40	Nurse	1	250	15
HP08	Centre	F	42	Nurse	16	250	15
HP09	Centre	F	59	Nurse	39	250	15
HP10	South	F	42	Psychologist	14	90	10
HP11	North	M	45	Physiotherapist	20	5	5
HP12	South	F	50	Nurse	2	N/A	Not applicable

FULL LIST OF QUOTES

Theme 1: Awareness of the transition

Categories	*When*	*How*	*Due to…*	*Patient's interview style*
Sub-categories	• Before the communication HPs (Ps, SOs, Ns)<br>• After the communication (P)<br>• Never happened, the patient is not aware (Ps, HPs)	• Suddenly (SOs)<br>• Difficulties in defining when the transition happened (Ps, Ns, HPs)	• Disease symptoms (Ps, SOs)<br>• Impact on activities of daily living/Loss of autonomy (Ps, SOs)<br>• No more remission (Ps, SOs)<br>• Worsening despite magnetic resonance imaging stability (Ps, SOs, Ns, HPs)<br>• Participation to this project (Ps, SOs)<br>• Changing or suspending disease modifying treatment (Ps, Ns, HPs)	• Tortuous (Is)<br>• Induce participant's answer (Is)<br>• Vague (Is)<br>• Verbose (Is)

__Category 1__**: When**

Sub-categories

Patient's awareness:

- **Before the communication (Ps, SO, Ns)**
  - P:
    - "So, eh, I have to anticipate that, mm, I was aware of the passage, because, eh, I had a lot of information about it. I read which the types of diseases are, and consequently, when I began to... to experience more problems, the slight worsening… in short, those symptoms that are described in the secondary progressive form… and also hearing the health care professionals speaking, in dribs and drabs about my changes… I understood I was moved to this form of illness. I got there gradually... And I did not initially perceive... When, instead, I saw that, progressively, precisely, as the term says, but slowly, I was starting having other vision problems, and problems in other organs, in other things, I realized I had entered this type of illness." [P04: man, 44 years, EDSS 6, North]
    - "Let's say I noticed, there was no need for someone to tell me." [P11: man, 47 years, EDSS 6.5, Centre]
  - SO:
    - "Yes, yes, we understood it by our own. He noticed he wasn't the same as before... it was quite sudden, not ... gradual." [SO03: wife, 42 years, North]
    -

- N:
  - “Very often it is the patients that say that [the transition to SPMS] to you. He says: "But, doctor, listen…. But in the end, what this treatment is doing for me? "So he already gives you almost the input of speaking about the transition.” [N02: woman, 59 years, North]

- **After the communication (P)**
  - “I wasn’t aware of having this progressive form... Compared to the worsening of the disease yes... yes... I have always spoken about it, but I never ascribed it to a degenerative form, because I did not know the difference between forms.” [P01: man, 46 years, EDSS 6.5, North]

- **Never happened, the patient is not aware (P, HP)**
  - P:
    - “I did not know even if they wrote it. Then I decided to let doctor supported me, I trust them and I have always have done what they told me to do. I did not ask for anything because when I knew I had multiple sclerosis, I panicked ... I don’t know where this situation will bring me, so I accepted to participate in a trial. I did not know what was that “SP”. I only looked the medical report that said the disease was still stable, there is only a small micro signal so, ok, let’s go on!” [P02: woman, 60 years, EDSS 4, North]
  - HP:
    - “It's another diagnosis, basically. Sometimes it is not even understood.” [HP05: woman, 50 years, social worker, North]

Category 2: How

Sub-categories:

- **Suddenly (SOs)**
  - “[The transition to SPMS] it was quite sudden, not... not gradual.” [SO03: wife, 42 years, North]
  - “It happened all of a sudden. She lost her autonomies.” [SO02: husband, 41 years, North]

- **Difficulties in defining when the transition happened (P, Ns, HP)**
  - P:
    - “Reading the medical reports, I understood that the resonances were always unchanged, and knowing how the disease evolves, I said: "Well, it's since '99 that I have it, maybe even before […], we are in 2017 ... » Did we expect to “skip the ditches”?!… I could expect it! ... down in black and white, I had read SP in 2012, more or less... written by a physician… But I said: "Oh well, he writed it ..." It has not affected me more than that. After that, I read it again one month ago, during another visit with Dr S. Every so often I have read “SP” or with “progressive outcome” or severly effected. So, it's really something that... um... I... I already knew, but down in black and white, oh well, the title of this study! Perhaps, if I have to think about an official communication, from the MS Centre, it is the participation in this study.” [P06: woman, 39 years, EDSS 7, North]

- Ns:
  - “As we know, it is difficult to define the passage, to a secondary progressive form.” [N07: woman, 34 years, North]
  - “No, it's difficult, you can’t give an answer.” [N02: woman, 59 years, North]
  - “In my opinion, the problem of the transition between RR and SP is basically ours [Neurologists]. In the sense that I cannot be sure when the patient becomes secondarily progressive. Even if, at a certain point, I begin to perceive it, I have the certainty only further on. So, it is something that requires we walk a specific path with the patient.” [N05: woman, 50 years, South]
- HP:
  - “It is very difficult to sanction this passage!” [HP05: woman, 50 years, social worker, North]

<u>Category 3:</u> **Due to…**

Sub-categories:

- **Disease symptoms (Ps, SOs)**
  - Ps:
    - “I began to notice it [the progressive component] in my walking difficulties.” [P05: woman, 44 years, EDSS 6, North]
    - “I felt more and more fatigability. Fatigue often forced me to stop. I have no energy to carry on my daily activities, I feel numbness on the left side of my body, difficulties in walking. It is a difficulty not easy to explain. I often fall [...] I lose my balance and find myself on the ground and then an effect of bladder torpor follows.” [P01: man, 46 years, EDSS 6.5, North]
  - SOs:
    - “He has just the physical thing, because he does not have pain. He has a lot of difficulties in moving, walking. Spasticity is also a big issue.” [SO03: wife, 42 years, North]
    - “A real break down happened after a lot of years. Everything stop working properly: the eye, the hand, the leg. And... he could not get to the bathroom, that is all an incredible collapse”, you feel helpless…” [SO04: wife, 49 years, North]

- **Impact on activities of daily living/Loss of autonomy (Ps, SOs)**
  - Ps:
    - “Before that, my life was normal. Now I cannot tie my shirt, and I have to wear t-shirts only, I cannot use lace in my shoes, only velcro shoes! There are also many more small things I cannot do anymore! I have also some difficulties in writing, it is hard to hold the pen; when I eat, sometimes the spoon falls down, I struggle in holding a glass. I discover all these difficulties gradually.” [P01: man, 46 years, EDSS 6.5, North]
    - “I was no more able to take care to my family needs, such as my child needs.” [P01: man, 46 years, EDSS 6.5, North]
  - SOs:

- “He is no longer able to do what he could do maybe a few days before. Spasticity gradually increased, one leg, the other… and then the arms. He also lost strength and grip in his hands.” [SO03: wife, 42 years, North]
- “Suddenly, she lost her autonomy in the daily activities. In six months we passed from a normal life, to not being able to go to the bathroom alone. She needs help in doing everything, dressing up, cooking, everything!” [SO02: husband, 41 years, North]

- **No more remission (Ps, SO)**
  - Ps:
    - “I have spent 5 days in the hospital taking steriods, doctors did a lot of procedures, but I gradually noticed that there was no remission, no recovery.” [P05: woman, 44 years, EDSS 6, North]
    - “I hope it was as in childhood attacks, that a little steroid would have given me the necessary relief to restore my normal life ... At the beginning, steriods gave me some benefit, but in more recent years It was totally ineffective.” [P01: man, 46 years, EDSS 6.5, North]
  - SO:
    - “At the beginning, everything was under control, he took the drugs. Occasionally there were some relapses, he needed to take steriods, but he did a normal life, cycling and everything. After a lot of years, a real break down happened. Everything stopped working properly: the eye, the hand, the leg. And... he could not get to the bathroom, that is all an incredible collapse”, you feel helpless... We wrote to the doctor, trying to find a solution, but there is no solution!” [SO04: wife, 49 years, North]

- **Worsening despite magnetic resonance imaging (MRI) stability (Ps, SO, N, HPs)**
  - Ps:
    - “My MRI is unchanged since 2008 and I think: Wow, it's unchanged and I get worse.” [P06: woman, 39 years, EDSS 7, North]
    - “I knew my MS was a RR type, but, my resonance shows no activity, but I can feel a slow worsening...It is only 8 months that I am using the wheelchair... I often asked my neurologist: How is it possible? My resonance is ok and I'm worsening.” [P15: man, 39 years, EDSS 7, South]
  - SO:
    - Someone told me: “if the MRI is stable and he is worsening it is not a good news. It means he is entering the progressive phase. He has no more relapses but everything is worsening. He is in the progressive phase.” [SO04: wife, 49 years, North]
  - N:
    - The patient typically says: "I do not understand why I'm getting worse. You told me that my MRI is stable, I have had no relapses, I'm taking the drugs, why do I get worse? "So, this is the core of the problem. [N07: woman, 34 years, North]
  - HPs:
    - “You understand that they (patients) are really lost because they report as positive note the fact that the MRI is stable, eve thought they add “I am feeling worse." This could also be assign of some difficulties in communicating with the neurologist.” *[HP01: woman, 47 years, psychologist, North]*

- “On one side the MRI is stable, on the other side the recongnize they are worsening.” [HP05: woman, 50 years, social worker, North]

- **Participation to this project (P, SO)**
  - P:
    - INTERVIEWER: Have you ever spoken about the SPMS with the neurologist? PATIENT: –No. INTERVIEWER: – Have doctors ever explain the term “SP” you found in the clinical report? PATIENT: No, never. I read it today with the neurologist in the informed consent form. It is since 2012 that the neurologist wrote it on my clinical records, but, to be honest, I simply look for “stable” and it is ok to me!” [P02: woman, 60 years, EDSS 4, North]
  - SO:
    - The communication of the transition to a SP for of the disease, arrived when my husband received the invitation to participate in this study. His reaction was: "The neurologist did some mistakes in sending me this e-mail! It's not for me.” [SO01: wife, 48 years, North]

- **Changing or suspending disease modifying treatment (P, N, HP)**
  - P:
    - “The neurologist have always told me everything: “You have this progressive form, I cannot propose you any kind of preventive drugs". He told me “You won’t be able to walk again”. [P13: woman, 42 years, EDSS 6.5, South]
  - N:
    - “The hardest thing, if the patient is doing an immunomodulatory therapy, is the management of the therapy: to understand if it makes sense to continue with a preventive therapy, or decide to stop it!” [N07: woman, 34 years, North]
  - HP:
    - “Patients recognize they entered in the progressive form when the stop all the preventive treatments!” [HP04: woman, 48 years, nurse, North]

Category 4: Patient’s interview style (All these aspects were reported by the interviewers).

Sub-categories:

- **Tortuous**
  - Sometimes it was hard to understand what the patient was saying. It was easy to lose patient’s train of thoughts! The style was tortuous and the person seemed confused. The sensation was that the topic wasn’t clear to him.
- **Induce participant’s answer**
  - In some interviews I had this strong feeling the person got lost, as if he or she was not aware of the having a SPMS. Hence, I tried to clarify what I was saying; I asked more specific questions, in order to be less vague. It only resulted in me suggesting the answer, but the feelling remained that the patient didn’t know what to say.
- **Vague**
  - Once I completed the interview, I was confused. Thinking about it or reading all the verbatim I was surprised of how vague was each patients’ answer.
- **Verbose**

- You can feel this person was not aware of the transition, because she could not stop "beating around the bush". They speak a lot, in a really verbose style, without the ability of reaching the target. It seems like she wanted to fill in the conversation, but with few awareness of the topic.

Theme 2: Communication of the Transition

Categories	*Dynamics*	*How communication is perceived*	*When neurologist speaks about SPMS*	*Patient's reaction to the communication*
Sub-categories	• Acknowledgment with the neurologist (Ps, SOs, Ns, HPs)<br>• Acknowledgment after patients request for more information (P, SO, Ns)<br>• Indirect (i.e. medical report) (P, HP)<br>• Prevented by the patients (Ps)<br>• Progression is not communicated (Ps, SOs)<br>• Ambiguous/Unclear (P, SO, N, HP)<br>• Open and clear (SO, HP)	• Important (P, SOs, Ns, HPs)<br>• Not important (Ps, Ns)	• Since the diagnosis of RRMS (N)<br>• Not at the moment of the diagnosis, but further on (Ns)	• Emotional<br>- Panic (P)<br>- Fear (P)<br>• Defensive strategies<br>- denial (P, SO, N)<br>- avoidance (Ps, N)<br>- displacement (P)<br>• Openness to experience (P)<br>• Acceptance (P)

<u>Category 1</u>: **Dynamics**

Sub-categories:

- **Acknowledgment with the neurologist (Ps, SO, Ns, HPs)**
    - Ps:
        - "When the neurologist told me about it. It was a only a confirmation of what I have already sensed." [P15: man, 39 years, EDSS 7, South]
        - "We concluded that the disease was turning from relapsing-remitting to secondary progressive MS. The doctor explained me SPMS it is not connected to active plaques. More than anything else it was an observation... of both... because, as I told you, the situation slow down... it got worse... and the resonance told you that the disease was stable, but it wasn't so under a clinical point of you. The EDSS passed form 5.5, to 6, and now we are at 7." [P15: man, 39 years, EDSS 7, South]
    - SO:
        - "We can't complain anyhing. The neurologist was direct and clear at each visits. We have always discussed each change in my wife's functioning"." [SO02: husband, 41 years, North]
    - Ns:
        - "It's a topic that is faces step by step. It never happens that you suddenly and clearly tell to a patient: "You have this form." So it is something that, in some way, matures

over time and, as my colleague said, it is the patient himself who leads you to this conclusion". [N07: woman, 34 years, North]
- We must take care of the patient considering all the different aspects. We need to value a comprehensive range of things, so as to progressively speak about this topic: "The treatment we are doing is no longer needed, maybe it gives you more side effects than benefits". But it is a long journey. To do it well, it is a long journey, it is a path that requires a quantity of resources that we will never have. [N05: woman, 50 years, South]

- HPs:
  - "To reach this awareness together (PT and HP) requires time… it is not an immediate process." [HP07: woman, 40 years, nurse, Centre]
  - "You need to slowly prepare the patient to receive this information. It is not easy. However, we have to do it. It is important to help the patient to feel that his/her life is not ended." [HP12: woman, 50 years, nurse, South]

- **Acknowledgment after patients request for more information (P, SO, Ns)**
  - P:
    - I had already understood, that's why I asked! "Doc, do you think I am progressive?" [P11: man, 47 years, EDSS 6.5, Centre]
  - SO:
    - We spoke with the doctor about what was going on. He told us: "Yes, the disease is progressing". *[SO03: wife, 42 years, North]*
  - Ns:
    - "Usually, I try to get to the heart of the matter, valuing patient's personal experience of symptoms worsening". [N01: man, 56 years, North]
    - Very often, it is the patient itself that brings the topic. "Doctor, what is the benefit of this therapy? What is it doing?". In some way, tha patient already provided you with the input to speak about the progression. [N02: woman, 59 years, North]

- **Indirect (i.e. medical report) (P, HP)**
  - P:
    - "He [the neurologist] wrote it in the clinical report but there has never been a specific communication on the passage." [P01: man, 46 years, EDSS 6.5, North]
  - HP:
    - "People come to me with the medical report, the term "secondary progressive" is there, but it is not really clear what it means." [HP05: woman, 50 years, social worker, North]
    -

- **Prevented by the patients (P)**
    - "I knew it [transition to SP]. I sensed something… and the doctor told me about it, but the reality is that I didn't want to understand it, to face the reality. I can't even speak about it!" [P05: woman, 44 years, EDSS 6, North]

- **Progression is not communicated (Ps, SOs)**
  - Ps:
    - I have never spoke about "SP" with my neurologists. [P02: woman, 60 years, EDSS 4, North]

- “We did not speak about what was going on.” [P03: man, 52 years, EDSS 6, North]
  - SOs:
    - “They did not tell you about what was going on. Nobody clearly states that you are entering in the progressive phase.” [SO04: wife, 49 years, North]
    - “They don’t answer, they don’t tell you anything. You need to reach this awareness by your own.” [SO03: wife, 42 years, North]
- **Ambiguous/unclear (P, SO, N, HP)**
  - P:
    - “I did not realize it… I only partially sensed it because I still have some difficulties in accepting my MS… During the last follow up visits the doctor told me about that, but because of my tendency to… it is hard for me to accept... so…” [P07: man, 49 years, EDSS 6, North]
  - SO:
    - “In our case, there was no explicit communication of the transition from a relapsing-remitting to a secondary progressive form.” [SO01: wife, 48 years, North]
  - N:
    - “It is something implicit. It is hard that it is clearly faced. Sometimes, we [HP] have some difficulties in stating that the patients has transitioned to a progressive form.” [N04: woman, 50 years, Centre]
  - HP:
    - “Patients often ask us for confirmation. Very often the neurologist writes something unclear about the progression in the clinical record. The passage to a secondary for is not emphasized, not clear, with the results that patients then ask to other health care professionals.” [HP08: woman, 42 years, nurse, Centre]
- **Open and clear (SO, HP)**
  - SO:
    - “I cannot blame the neurologist. On the contrary, he was very open, he told us: "We are going backwards. We cannot do anything about it. MRI weaks. Blood tests. Let's try to change the treatment. He tried everything! He was always very open, and easy to reach.” [SO02: husband, 41 years, North]
  - HP:
    - “[...] Other times the neurologist communicate this passage in a very direct way and he/she clearly explain the transition!” [HP08: woman, 42 years, nurse, Centre]

<u>Category 2</u>: How communication is perceived

Sub-categories:

- **Important (Ps, SO, Ns, HPs)**
  - Ps:
    - “Surely knowing it and being aware of the period I was going to live, receiving this information from an authoritative external source, would have given me a little more comfort.” [P01: man, 46 years, EDSS 6.5, North]
    - “Yes, it would have helped me to be aware of the moment I was living, I wondered and think about a lot of things by my own... I was confused "But it will always be... maybe yes... maybe not... what am I going to deal with?" I was worried about the future, especially because I felt alone in dealing with it… a mountain bigger than you!” [P01: man, 46 years, EDSS 6.5, North]

- SO:
  - "I wanted to know more about it!" [SO04: wife, 49 years, North]
- Ns:
  - "Nowadays, considering the possible side effects of some drugs, it is even more important to stop treatment when they are no more effective. Therefore, it is even more important to communicate what is going on. For this reason, I am start sensing it is really important to discuss this topic with the patient. It may change the therapeutic approach we use!" [N05: woman, 50 years, South]
  - "I believe it is useful and people deserve to know about it." [N03: woman, 49 years, Centre]
- HPs:
  - "A confrontation with the neurologist is important and it must happen, sooner or later! Patients asked us about their neurological condition, very often. They seems in difficulties and they are looking for answers. They have the right to know. I am aware there is this communication problem. A lot of patients in rehabilitation have great and unrealistic expectations. Despite they are already severely affected, maybe they are on the wheelchair; they are totally unaware about the progression. For these reasons, sometimes patients question the rehabilitation program they are doing: "I can't understand why the physiotherapist doesn't make me work on my legs, or why they do not make me stand! This confusion depends on the lack of information and communication about the disease progression." [HP11: man, 45 years, physiotherapist, North]
  - "It is important to provide patient with a comprehensive explanation, let him/her imagine his/her future. In some way, to train the patient." [HP12: woman, 50 years, nurse, South]

- **Not important (Ps, Ns)**
  - Ps:
    - "No, it should not be too explicit, otherwise it is like saying: you are dying No. It would be too much!" [P06: woman, 39 years, EDSS 7, North]
    - "Let the person being not aware. Do not insist to provide him/her information." [P05: woman, 44 years, EDSS 6, North]
  - Ns:
    - "Patients want to be followed. This is the important thing! In my opinion, there is no need to inform about the type of the disease." [N02: woman, 59 years, North]
    - "It [to know about the progression] is devastating for them, so we are all happy if we do not face it. The patient does not have to deal with it... It's a complex thing, I would not let us to be guided too much by the classification." [N01: man, 56 years, North]

<u>Category 3</u>**: When neurologist speaks about SPMS**

Sub-categories:

- **Since the diagnosis of RRMS (N)**
  - "Usually, I explain immediately, at the moment of the diagnosis, that multiple sclerosis has an inflammatory phase and a degenerative phase." [N07: woman, 34 years, North]

- **Not at the moment of the diagnosis, but further on (Ns)**
    - "It is not so frequent that I address the topic with the patient." [N01: man, 56 years, North]
    - I hardly talk to a patient about what will happen to his/her in the future [...]. It happens to me to answer to some specific questions, only with patients who have already experienced the disease with their relative. "Now I am in this condition. When will I be like my mother, my father, my sister?" This is the only time I am faced with this kind of .. [N02: woman, 59 years, North]
    - "I have some resistances to explain that there is "phase A and Phase B", immediately at the beginning. I do not agree so much with these classifications. It is obvious that there is a degenerative component is obvious, but when there is a 20-year-old girl in front of us, I believe this is not the main topic! You work on that further on and with the adequate time." [N01: man, 56 years, North]

<u>Category 4</u>**: Patient's Reaction to the communication**

Sub-categories:

- **Emotional (P)**
    - **Panic**
        - There was a moment in which I thought: "Ok, that's it!" [I have a progressive form]. I almost panicked. [P06: woman, 39 years, EDSS 7, North]
    - **Fear (P)**
        - "One important aspect of the new diagnosis [SPMS] is fear, the fear of not being able to recover anymore." [P06: woman, 39 years, EDSS 7, North]
- **Defensive strategies**

Denial (P, SO, N)

- P:
    - I knew it [transition to SPMS]. I sensed something… and the doctor told me about it, but the reality is that I didn't want to understand it, to face the reality... I can't even speak about it!" [P05: woman, 44 years, EDSS 6, North]
- SO:
    - "When the neurologist invited my husband to participate in this study the first reaction was: It is a mistake! I will write him that he is wrong, it is not true!" [SO01: wife, 48 years, North]
    -
- N:
    - "The patient, even the very educated one, is not always willing to understand the situation. It's a psychological reaction." [N04: woman, 50 years, Centre]

Avoidance (Ps, N)

- P:
    - They told me to do some physiotherpay. I did it for a while, but then I stopped. I was afraid of putting all my efforts and not obtaining any improvements. For this reason, I decided not to try, because I would have anticipated the wake up with reality." [P05: woman, 44 years, EDSS 6, North]

- I deny it and go on [...] then I block, block the emotion… I do not know if this strategy helps me. [P06: woman, 39 years, EDSS 7, North]

o N:

- "It [to know about the progression] is devastating for them, so we are all happy if we do not face it. The patient does not have to deal with it... It's a complex thing, I would not let us to be guided too much by the classification." [N01: man, 56 years, North]

Displacement (P)

- "I tried not to think about it and pay attention to other thing. For example, I have some walking difficulties, but it is because the shoes bother me." [P05: woman, 44 years, EDSS 6, North]

- **Openness to experience (P)**
  - I said: "No! I try anyway, we will see how it goes." [P06: woman, 39 years, EDSS 7, North]

- **Acceptance (P)**
  - This summer has been really hard. For this reason I had to face the reality. "Ok, you know it is in this way. Admit it! This is the reality" [P06: woman, 39 years, EDSS 7, North]

Theme 3: Dealing with symptoms worsening

Categories	***Personal Experience***	***Adjustment strategies***	***Difficulties in adjustment***
Sub-categories	• Anxiety (P)<br>• Anger (P, SOs)<br>• Guilt (P, SO)<br>• Fear (P, SO, HP)<br>• Sadness (Ps)<br>• Loneliness of both patients and SOs (Ps, SOs)<br>• Humiliation/shame (Ps, HPs)<br>• Disappointment (P, SO)<br>• Helplessness/powerlessness (SO, Ns, HPs)<br>• Nervousness of both patients and SOs (SOs)<br>• Frustration (Ns)<br>• Hope (HP)<br>• Confused/unprepared (SO; HPs)<br>• Worry (HP)<br>• Shame (HP)	*Concrete solutions*<br>• Surfing the web (Ps, SO)<br>• Looking for new drugs (Ps, SO)<br>• Autonomy maintenance (Ps, SO)<br>• Work maintenance (P, SOs)<br>• Planning activities carefully (one activity at time) (Ps)<br>• Self-care (Ps)<br>• Staying in contact with the neurologist or HPs (P, SO, N, HP)<br><br>*Inner resources*<br>• Tenaciousness (P,)<br>• Grounding (P)<br>• Acceptance (Ps)<br>• Meaning (P, SO)<br>• Mindfulness (carpe diem) (SO)	*Inner factors*<br>• Giving up (Ps, HP)<br>• Not sharing (Ps)<br>• Retirement from the relationship (P)<br>• Cognitive fusion (P)<br>• Passiveness (HP)<br>• Experiential avoidance/denial (P, SO)<br><br>*Environmental factors*<br>• Uncertainty (P, SO)<br>• Presence of children (SOs)

		• Being in contact with personal values (HPs)<br><br>*Spirituality*<br>• Religion/faith (P)<br><br>*Social connectedness*<br>• Social support (Ps)	

<u>Category 1</u>: Personal experience

Sub-categories:

- **Anxiety (P)**
  - “Oh yes, I felt so anxious!” [P06: woman, 39 years, EDSS 7, North]

- **Anger (P, SOs)**
  - P:
    - I get angry. Just an example, I need a pen, so I say, "Ok now I stand up and go. Now I stand up and go.” However, at the end, I can’t stand up and I can’t go! So I have to ask someone, and I start feeling so angry! [P06: woman, 39 years, EDSS 7, North]
  - SOs:
    - “He was always angry, very nervous!” [SO05: wife, 51 years, North]
    - “His anger is that there is no treatment available!” [SO03: wife, 42 years, North]
    - “In the recent years and during my father worsening, the relationship in my family were really characterized by anger. A livid anger, triggered by all the small things.” [SO06: daughter, 27 years, North]

- **Guilt (P, SO)**
  - P:
    - “I have tried to take care of me, but then I felt guilty for all the things I failure… It’s my fault! I could do more…” [P05: woman, 44 years, EDSS 6, North]
  - SO:
    - “I feel guilty.” (SOs’ sense of guilty) [SO04: wife, 49 years, North]

- **Fear (Ps, SO, HP)**
  - Ps:
    - “I was totally afraid!” [P15: man, 39 years, EDSS 7, South]
    - “What I am experiencing is pure fear! It is a deep fear! How can I go to work? How can I reach a certain place? It is a fear about how to do practical things.” [P04: man, 44 years, EDSS 6, North]
    - “I am afraid of the future, especially when the future is like a mounten, absolutely bigger than you!” [P01: man, 46 years, EDSS 6.5, North]
    - “I have this feeling that if I spend too much time seated I will not walk anymore!” [P06: woman, 39 years, EDSS 7, North]
  - SO:
    - “I am scared to harm him while I am helping him. I am really afraid about that.” [SO05: wife, 51 years, North]
  - HP:

- “It is true there is hope, but there’s also a lot of fear!” [HP08: woman, 42 years, nurse, Centre]

- **Sadness (Ps)**
    - “I lost my autonomy, this makes me really sad!” [P15: man, 39 years, EDSS 7, South]
    - “Right now, speaking aobut it, a drop fals down on my cheek.” [P05: woman, 44 years, EDSS 6, North]

- **Loneliness of both patient and SOs (Ps, SOs)**
    - Ps:
        - “I have spent many years without telling anybody about it, without saying it to myself.” [P13: woman, 42 years, EDSS 6.5, South]
        - “You feel alone.” [P11: man, 47 years, EDSS 6.5, Centre]
        - “The result was that I have managed all this worsening all alone.” [P01: man, 46 years, EDSS 6.5, North]
    - SOs:
        - “I feel lonely, because basically... we do not have other relatives here. Facing this situation is a bit difficult.” [SO05: wife, 51 years, North]
        - “We felt alone, totally alone!” [SO06: daughter, 27 years, North]
        - “I feel lonely! Friends invite you somewhere, let’s say on the river. Once you reach the place you discover that it is totally inaccessible. They did not take into consideration your difficulties!” [SO01: wife, 48 years, North]

- **Humiliation/shame (Ps, HP)**
    - Ps:
        - “The most humiliating aspect of the disease is to not be able to take care of my son, to play with him, supporting him while is grouwing up. I cannot play with him as the other dad does. I cannot give a horseback ride to my son. My wife has a huge burden. I have to step aside very often, this is humliating!” [P01: man, 46 years, EDSS 6.5, North]
        - “I was ashamed!” [P13: woman, 42 years, EDSS 6.5, South]
    - HP:
        - “Some patients face the disease only when it becomes secondary progressive without telling anyone about it. They feel so ashamed of having this illness.” [HP10: woman, 42 years, psychologist, South]

- **Disappointment (P, SO)**
    - P:
        - “It is a pity that even the symptomatic drugs have no effect on me. It is really disappointing!” [P01: man, 46 years, EDSS 6.5, North]
    - SO:
        - “It is extremely disappointing not to see any results. You do whatever the doctor says, you put a lot of efforts, but you never obtain any results! My husband knows that he will never be as before, but he would like to have a decent life! So it is really disappointing, you can do everything, but nothing changes!” [SO03: wife, 42 years, North]

- **Helplessness/powerlessness (SO, Ns, HPs)**
    - SO:

- "I think the right word to describe what you feel when there is the transition to a secondary progressive form is helplessness. You feel totally helplessness. You are no more able to reach the toilet, you can't do anything. So it starts the helplessness, and you start fighting to find a solution. It never comes." [SO04: wife, 49 years, North]

  - Ns:
    - "It's a feeling of helplessness… As my collgue [N05: woman, 50 years, South] said, I also feel the lack of everything… of all the services." [N06: woman, 32 years, South]
    - "The point is that [when the progression start] you don't have perspectives… you loose your role as neurologist." [N03: woman, 49 years, Centre]
  - HPs:
    - "The feeling of helplessness… it's the worst part." [HP06: man, 46 years, psychologist, North]
    - "Neurologist's helplessness. In my opinion, neurologists sometimes feel helpless because their intervention is really focused on drugs." [HP05: woman, 50 years, social worker, North]

- **Nervousness of both PT and SOs (SOs)**
    - "He [the patient] was very often upset and nervous..." [SO05: wife, 51 years, North]
    - "I feel bad too... I have the child to take care of… I get nervous too!" [SO03: wife, 42 years, North]

- **Frustration (Ns)**
    - "As neurologist, I also feel the frustration of taking care of a person with a progressive form. If you have to deal with a person with relapsing remitting you can use a lot of therapeutic options. This is so much more gratifying than dealing with a progressive form!" [N05: woman, 50 years, South]
    - "It is frustrating. At the moment of the diagnosis, when it is a RR form you can choose between lots of different drugs. When there is the transition to a progressive form, the patient asks you: "ok, what can we do now? Which kind of mediaction do you suggest?" Now we have very few option! Hence, we start using symptomatic treatments. This change in the management of the person has an impact on both the patient and us. We need to deal with both these sides. Even though the patient has already learnt that he/she can become progressive, because you have told him/her a lot of times, that this could happen, it is really hard when you really have to deal with that because you are without "tools/instruments". [N04: woman, 50 years, Centre]

- **Hope (HP)**
    - I had the feeling that in young people, after discouragement then comes hope, a glimmer of hope. In fact, they say: "There is the research… maybe, in the future new therapy will come out." [HP04: woman, 48 years, nurse, North]

- **Confused/Unprepared (SO; HP)**
  - SO:
    - "A person feels totally unprepared in this context." [SO04: wife, 49 years, North]
  - HP:
    - You can notice they [the patients] are disoriented and that there is some difficulties in the communication with the neurologist on this topic because they refer to the

fact that the MRI is stable, as positive thing. This is considered as positive even thought “I am getting worse”. Thay said something like that: “the MRI is stable, but I am feeling worse”, or “the neurologis could doubt about my perception becuse I am getting worse despite MRI stability”. It seems they are missing the point, they did not understand there are also other things they should monitor. It is really a critical moment. [HP01: woman, 47 years, psychologist, North]

- **Worry (HP)**
  - Sometimes the patient says: “I'm worried, I don’t know, the doctor told me about the walking stick… [HP03: woman, 56 years, physiotherapist, North]

- **Shame (HP)**
  - “There is another kind of patients who start dealing with the disase only when it become secondary progressive. They usually have hidden the disease to their friends, with the exception of the closest relationships. Hence, they can feel ashamed of suddenly communicating that it is not hernia, but something more severe… it is the same in the working setting.” [HP10: woman, 42 years, psychologist, South]

<u>Category 2</u>: Adjustment strategies

Sub-categories:

<u>Concrete solutions</u>

- **Surfing the web (Ps, SO)**
  - Ps:
    - “I usually surf the web, read the forum, I do my best to keep me informed about new drugs…” [P11: man, 47 years, EDSS 6.5, Centre]
    - “I was a little confused… I didn’t know… I started to look for information on internet in order to see if I could find a possible, alternative cure.” [P01: man, 46 years, EDSS 6.5, North]
  - SO:
    - “You feel powerless, you start fighting and searching on the web, on a newsletter.”[SO04: wife, 49 years, North]

- **Looking for new drugs (Ps, SO)**
  - Ps:
    - We are on hold […] waiting for the Doctor to say: “Massimo, there is this new drug” or “Massimo, we are studying…” for example we are waiting for the stem cell. We know that it will take long… three years at least! [P11: man, 47 years, EDSS 6.5, Centre]
    - “I often ask if there are new drugs available.” [P01: man, 46 years, EDSS 6.5, North]
  - SO:
    - “So you're always looking for a new drug… which is never available.” [SO04: wife, 49 years, North]

- **Autonomy maintenance (Ps, SO)**
  - Ps:
    - “If my husband is away on a business trip, my mom stays with me and sleeps with me. In this way, I am not alone if my daughter needs something or I have to weak up during the night. Of course, my mom can’t pick up me and I can keep working on

my automomy, even when she stays at our place." [P06: woman, 39 years, EDSS 7, North]
    - "When I prepare the meal I can be a little be unbalanced. No worries, I put all the ingredients on the table, I sit down and keep going… same thing with ironing." [P02: woman, 60 years, EDSS 4, North]
- SO:
    - He can't do that, but if I try to say something like: "I give you a lift and I can pick you up when you have finished", his reply is "Absolutely not!" He doesn't want me to pick him up everytime. I can only convince him when it is raning. He uses the motorbike, he tries very hard… it is a huge efforts, but he can bear to loose his own autonomy. He wants to be free to go out for lunch with his colleagues, to go to the bar, to move around. If you take this off from his it is like taking everything off from him. [SO01: wife, 48 years, North]

- **Work maintenance (P, SO)**
  - P:
    - "I have always tried to keep my job, to never leave it, because it is a very important aspect of my life. My job is my anchor to the reality, to who I was before." [P01: man, 46 years, EDSS 6.5, North]
  - SO:
    - His work provide him with this sense of autonomy… I also prefer that he goes to work. Can you imagine if he would spend all the day at home with me?! I am sorry that is a huge effort for him to reach the working place, but in a way he keep himself busy! During the working hours, his mind is really committed to the working activities. I found it really positive. It provides him with a purpouse. In particular, he thinks, "I have to do that because I have to financially support the family. His aim is to financially support the family". [SO03: wife, 42 years, North]

- **Planning activities carefully (one activity at time) (P)**
  - P:
    - I learned to focus on one activitiy! For example, I could say: "today I cook everything by myself!" It becomes my main activity that day! [P06: woman, 39 years, EDSS 7, North]

- **Self-care activities (P):**
  - P:
    - I think every person need to dedicate time to him/herself! Such moments make you feel a sense of achievement. I found this moments in religion and going to the gym. These two committments help me hugely! [P01: man, 46 years, EDSS 6.5, North]

- **Staying in contact with the neurologist or HPs (Ps, SO, N, HP)**
  - Ps:
    - "Above all, the most important thing is that if I had a doubt I can write to the neurologist and receive an answer." [P06: woman, 39 years, EDSS 7, North]
    - "I am under Dr. XX's care. He is always available, this is foundamental! To be able to rely on someone when you need that, it's crucial! It is not in terms of physical presence, sometimes there is nothing practical he can do, but to know there is someone you can rely on… someone with which you can discuss your situation, this is the most precious thing." [P07: man, 49 years, EDSS 6, North]

- SO:
  - “The neurologist called each week to know how things were going. I can blame anybody. They all have done a great job! We have been very lucky. Despite that, this disease has freely progressed.” [SO02: husband, 41 years, North]
- N:
  - “The patient needs to feel that the neurologist takes charge of him/her, that they discuss and share all the changes in his/her functioning.” [N02: woman, 59 years, North]
- HP:
  - “Patients call and come to the MS centre every so often! They really need to stay in contact with us.” [HP12: woman, 50 years, nurse, South]

Inner resources

- **Tenaciousness (P)**
  - “You have to keep going no matter what! Whatever is going to do, you need to try.” [P02: woman, 60 years, EDSS 4, North]

- **Grounding (P)**
  - “I fell like in the middle of a storm, you try to rely on something that can support you, an anchor for the future. While dealing with my ’physical storm’ I looked for some pillars that supported me, such as my job, my faith and my son. These were my three elements.” [P01: man, 46 years, EDSS 6.5, North]

- **Acceptance (Ps)**
  - “It is important to recognize that it is not ’the end of the world’ and accept the limits I have.” [P06: woman, 39 years, EDSS 7, North]
  - It has been a while since I started perceiving a feeling of resignation. Something like “this is my life, even though my projects for the future were different”. It is what it is, hence I am trying to live my life in the best way possible. [P15: man, 39 years, EDSS 7, South]
  - Acceptance is really important. It implies a sense of proactiveness. Acceptance means being aware of the situation and it s crucial to be effective in taking any decision. What can I do if I need to go from A to B and I am encountering some barriers. I can be stuck somewhere, or I can accept it and seeking a way to overcome it, maybe walking aroung the “stone”, going back and taking another road, finding other strategies. I know I have the tendency to avoid thing but, in the end, you have to deal with what is going on. [P07: man, 49 years, EDSS 6, North]

- **Meaning (P, SO)**
  - P:
    - “The only positive thing in this moment is that you realize what really means to you in life! What are the really important things.” [P01: man, 46 years, EDSS 6.5, North]
  - SO:
    - “I am sorry because I know it is very hard for him, but it is really important that he keeps going to work. Work gives him a purpose in life. I don’t work, so he has to support the economy of the family. It provides him with a real meaning in life.” [SO03: wife, 42 years, North]

- **Mindfulness (carpe diem) (SO)**

- I wish I knew what will happen, but, in the end, I think "Carpe Diem"… to stay in contact with the present moment it is probably very useful. [SO01: wife, 48 years, North]

- **Being in contact with personal values (HP)**
  - "A patient of mine, 55 years old, needed a stick. She refused to use it because she couldn't accept it, but once she became granma everything changed. She is now happily using the stick because in this way she can take are of her nephews and nieces." [HP04: woman, 48 years, nurse, North]

<u>Spirituality</u>

- **Religion/faith (P)**
  - "I have found some relief in religion; I always had a good relationship with God. The church has been my only outlet." [P01: man, 46 years, EDSS 6.5, North]

<u>Social connectedness</u>

- **Social support (P)**
  - "Having many friends helped me a lot! Telling them about my state and emotions helepd me a lot!" [P06: woman, 39 years, EDSS 7.0, North]

<u>Category 3</u>: Difficulties in adjustment

Sub-categories:

<u>Inner factors</u>

- **Giving up (Ps, HP)**
  - Ps:
    - "I just want to leave everything and go in a hospital and stay quiet there." [P01: man, 46 years, EDSS 6.5, North]
    - "The most important thing in life is don't put yourself down. I felt and rise so many times… you really need to fight in life." [P03: man, 52 years, EDSS 6, North]
    - "I have this feeling of resignation… even though my projects were different, this is the life I have." [P15: man, 39 years, EDSS 7, South]
  - HP:
    - "The elderly man usually says: 'I know it would have been this way.' What else can I do?" [HP04: woman, 48 years, nurse, North]

- **Not sharing (Ps)**
  - "I'd rather to do it my self. Others would't understand." [P10: man, 41 years, EDSS 4.5, Centre]
  - "I didn't share it [the transition to SMSP] with almost anybody. I didn't have the opportunity to speak about it with anybody, neither with medical doctor nor relatives." [P01: man, 46 years, EDSS 6.5, North]

- **Retirement from the relationship (P)**
  - "I withdrew from the world. My life is now the PC, the TV and playing with crosswords. Sometimes my daughter visits me but it is no more the same. Also about meeting other people, it is so rare!" [P03: man, 52 years, EDSS 6, North]

- **Cognitive fusion (P)**

- "I have this feeling that if I spend too much time on the wheelchair, I wouldn't never walk anymore! So if I have to do something, and I need the wheelchar to move, I keep having this thought and I remain stuck! It is nonsense because I simply trew away two hours thinking about it when I could do this in a couple of minutes!" [P06: woman, 39 years, EDSS 7, North]

- **Passiveness (HP)**
  - "Passivitity, this is the aspect on which we should work with patients." [HP06: man, 46 years, psychologist, North]

- **Experiential avoidance/denial (P, SO)**
  - P:
    - "Well, it is like I said 'this is better, I have not to take any drugs… I don't want to think about the disease'. I still avoid thinking about this problem. This is true also with other people, I am doing my best to hide it to others." [P12:woman, 56 years, EDSS 6.5, South]
  - SO:
    - "He has always denied the disease. At the beginning it was really disarmingly. He never went to pick up the MRI. The strongest denial I have ever seen. However, because of during the last period he started walking with the stick and he has bladder and incontinence problems, he now is dealing a bit more with the disease… but still, he denies whenever it is possible." [SO01: wife, 48 years, North]

<u>Environmental factors</u>

- **Uncertainty (P, SO)**
  - P:
    - "It would have been of help to be more informed and aware of what was going on. It would have helped me in not being alone in this process. I wondered will it be in this way forever? Will it be worse? What I am going towards in my life? I really would appreciate to receive more information!" [P01: man, 46 years, EDSS 6.5, North]
  - SO:
    - "Yes, they have more information, more clinical experience and evidences… but this disease is really unpredictable! This unpredictability and uncertainty it drives me crazy! I would prefer to know he will be on a chair and to know his future limitations than staiyng in this uncertainty." [SO01: wife, 48 years, North]

- **Presence of children (SOs)**
  - "It is really hard! The hardest situation is when you have children, particularly adolescence!" [SO04: wife, 49 years, North]
  - "You really feel powerless, you don't know what to do! It is not only about my husband, I also have to think about my son! When I think about all this I really have the feeling my word is falling apart!" [SO03: wife, 42 years, North]

Theme 4: Needs

Categories	***Met needs***	***Unmet needs***
Sub-categories	*Organization and management*<br>• Multidisciplinary equipe/holistic approach (N, HP)<br>• MS specialists (i.e. urologist, psychologist) (Ns, HPs)<br>• Neurologist availability (Ps, SO, N)<br>• Physiotherapy (P, SO, N, HP)<br>• Connection with AISM (P, SO, N, HP)<br><br>*Empowerment Training*<br>• Patients training (i.e. nutritionist, how to deal with daily life, hints for searching information) (N)<br><br>*Information*<br>• Easy access to information about aids (P, SO)	*Organization and management*<br>• Ad hoc plan of follow up visits with the neurologists and other HPs (P, Ns, HP)<br>• Multidisciplinary care/holistic approach (P, SO, Ns, HPs)<br>• Patient and SOs participation in multidisciplinary care (SO, Ns, HPs)<br>• A complete service charter of the MS center (HP)<br>• Home visits/care (P)<br>• Fast and easy access to MS center and its professionals (P, SO, N, HP)<br>• A case manager (Ps, SOs, HPs)<br>• Rehabilitation: More physiotherapy/occupational therapy (Ps, SOs, Ns)<br>• Collaboration with the general practitioner(HPs)<br>• Connection with AISM (SOs)<br>• Psychological support for both patients and SOs (individual, couple, family or group setting; homogeneous (SPMS) group discussion for both patients and relatives (Ps, SOs, Ns, HPs)<br>• Self-help group (social networking; communication beyond self-help group e.g. MS café; "Happy hour for sharing" experience (only SOs permitted) (P, SOs)<br>• Psychological support for HPs (Ns)<br><br>*Empowerment Training*<br>• Patients (i.e nutritionist, how to deal with daily life, hints for searching information) (Ns)<br>• HPs<br>- Communication (Ps, N)<br>- National and international cross training on SPMS (Meetings with multidisciplinary team to learn how to deal with daily living activities - evidence based, up to date information about symptomatic treatments; lifestyle, disease modifying treatments) (SO, Ns, HPs)<br>• SOs (i.e. disease and patient management; how to deal with daily life; how to deal with children in case one of the parents has SPMS) (P, SOs, Ns)

		Information<br>• Information on SPMS for patients and SOs (Ps, SOs)<br>• Clear information on non-pharmacological approaches and treatment/lifestyle/aids (P, SOs, N, HPs)<br>• Social care information (i.e. L.104) (SO, N)<br>• Website (or other sources) to share solutions and information (P, SOs)<br><br>*Policies*<br>• Job outplacement (P, SOs)<br>• Improving the social security net (Ps, HPs)<br>• Facilitating bureaucracy (P, SO)<br>• Supporting patient's autonomy/accessibility (P, SO, HPs)<br>• Developing specific national guidelines for people that are experiencing transition from RR to SPMS (HP)

<u>Category 1</u>: Met needs

Sub-categories:

<u>Organization and management</u>

- **Multidisciplinary equipe/holistic approach (N, HP)**
  - N:
    - "The medical treatment is important, but there are a lot of other interventions that are crucial, such as the psychological support! It is like the cake has the same ingredients, but the slice may change in size! When a patient is going towards progression, I activate other interventions based on patient's need, before stopping the disase modifying treatment." [N02: woman, 59 years, North]
  - HP:
    - "We are really lucky because we have the opportunity to support the patients by activating a lot of different HPs, such as the physician, the psychologist, creating connection with AISM. Not all the MS centers have this opportunity". [HP04: woman, 48 years, nurse, North]

- **MS specialists (i.e urologist, psychologist) (Ns, HPs)**
  - Ns:
    - "We have all the services available for chronic patients. There is the rehaiblitation service, the consultant urologist… etc... It is crucial to be able to activate all these specialists." [N02: woman, 59 years, North]
    - "We need to activate all the different MS specialists that a person may need to consult. Bladder problems, asthenia, or mobility issues, driving licence administrative stuff, may represent big problems in people life! We need to support

them in all these aspects. These are the practical consequences of neurological impairments.” [N02: woman, 59 years, North]

- HPs:
  - “It’s possibile to have a low moment???, but we are really well supported by two psychologists that are part of the team! Activating them is really important!” [HP04: woman, 48 years, nurse, North]
  - “Group setting interventions are really useful!” [HP10: woman, 42 years, psychologist, South]

- **Neurologist availability (Ps, SO, N)**
  - Ps:
    - “It is particularly important to me that, whenever I need, I can write to my neurologist and receive a reply!” [P06: woman, 39 years, EDSS 7.0, North]
    - “I really sing the praises of my neurologist! He is always available! This is really crucial, I can always count on him if I need him! Even if he cannot solve the problem, because there is no solution, to know that he’s available and willing to help is a big thing! You can alwys count on him!” [P07: man, 49 years, EDSS .06, North]
    - “It has been very helpful to me to come here and speak with the doctor: it is a real outlet for me. Maybe it doesn’t change the reality, because there is nothing you can do, but it makes you feel cared for!” [P02: woman, 60 years, EDSS 4.0, North]
  - SO:
    - “The neurologist called every week to know how things were going. He was really present and careful! I can’t complain anything!” [SO02: husband, 41 years, North]
  - N:
    - “I think the patient needs you caring for her/him. You have to discuss and share with her/him each step, each physical change and symptoms.” [N02: woman, 59 years, North]
- **Physiotherapy (P, SO, N, HP)**
  - P:
    - “Physiotherpy is crucial! The intensive rehabilitation is always something that helps in dealing with new difficulties and limitations you encounter during disease progression. Every time I went there I found it really beneficial at both physical and mental level.” [P07: man, 49 years, EDSS 6.0, North]
  - SO:
    - “Physiotherapy is the key! The pity is that it is never enough! These patients would need continuous rehabilitation. I can see the improvements my wife gains, but they are also lost very quickly!” [SO02: husband, 41 years, North]
  - N:
    - “Physiotherapy is always a great resource we activate for the patients, even before the transition to progression.” [N02: woman, 59 years, North]
  - HP:
    - “We work with many people with SPMS, physiotherapy is very often part of a multidisciplinary approach. Enhancing and promoting participation is one of our goals, also with people with SPMS.” [HP11: man, 45 years, physiotherapist, North]

- **Connection with the Italian MS Society, AISM (P, SO, N, HP)**
  - P:

- "AISM has been a great discovery! It is not only about fundraising, they really help you!" [P04: man, 44 years, EDSS 6.0, North]

- SO:
  - "We have been so lucky! We met a person from AISM and started to be connected with the association! This has helped us so much!" [SO02: husband, 41 years, North]
- N:
  - "One important thing is to create a good connection between the patient, his/her family and AISM. I think this may help them hugely! In several case, we contact AISM social workers." [N01: man, 56 years, North]
- HP:
  - "It is crucial for patients to be in contact with AISM. We try to create the connection if they haven't already developed one." [HP04: woman, 48 years, nurse, North]

<u>Empowerment training</u>

- **Patients training (i.e. nutritionist, how to deal with daily life, hints for searching information) (N)**
  - "We organize monthly meeting on a variaty of topics such as nutrition and lifestyle!" [N02: woman, 59 years, North]

<u>Information</u>

- **Easy access to information about aids (P, SO)**
  - P:
    - "Everytime I can't do something I look for some aids, tools or strategies that let me do the things I did before!" [P06: woman, 39 years, EDSS 7.0, North]
  - SO:
    - "Because of my job, I travel a lot and I can easily have access to more information about which aids are used in different countries. We found this amazing aid that helps her walking and that is not available in Italy! On the contrary, it was very easy to find it in the UK." [SO02: husband, 41 years, North]

<u>Category 2</u>: Unmet needs/resources

Sub-categories:

<u>Organization and management</u>

- **Ad hoc plan of follow up visits with the neurologists and other HPs (P, Ns, HP)**
  - P:
    - "Yes, in my opinion, it's an important thing. Because if I have to have a consultation and it is only possible after two months, at that time the consultation can be useless (e.g. the symptom has disappeared)" [P03: man, 52 years, EDSS 6.0, North]
  - Ns:
    - "These patients should have a dedicated path, which differs from the outpatient performance. If I prescribe my patient a consultation with a physiatrist, it can take up to six or seven months with the public healthcare system." [N07: woman, 34 years, North]
    - "Paradoxically, it would serve to increase the number of consultations, in these patients transitioning to SP. That is, unfortunately, the local guidelines for these patients... are lacking." [N05: woman, 50 years, South]

- HP:
  - “Sure, and at this stage they explain you, they tell you that they have difficulty when walking, they have sphincter problems, relationship with the partner is a problem… all these things come out only if you see the patient every 15 days, only with enduring relations…. So they come to the center less for consultations, but they need this contact with the therapist, they are more open, send messages via Facebook...” [HP08: woman, 42 years, nurse, Center]

- **Multidisciplinary care/holistic approach (P, SO, Ns, HPs)**
  - P:
    - “[…] and you know that the physiotherapist or the physiatrist can talk to your neurologist. That is, the neurologist, the psychologist, the psychotherapist, the physiatrist, the physiotherapist... talk to each other about you, using a scientific and rational language, different from the lay language of patients.” [P06: woman, 39 years, EDSS 7.0, North]
  - SO:
    - “What we need is a holistic approach.” [SO04: wife, 49 years, North]
  - Ns:
    - “We must follow the patient, as he rightly said, in the round.” [N05: woman, 50 years, South]
    - “The idea is that we probably have to build something really multidisciplinary from the beginning, starting when the patient is quite well.” [N01: man, 56 years, North]
  - HPs:
    - “Instead, approaching the patient sensitively, giving him hope despite all the complications that there may be in the evolution of the disease, in its chronicity... A multidisciplinary approach with the neuropsychologist, the speech therapist, the physiatrist, the physiotherapist, the nurse…” [HP07: woman, 40 years, nurse, Center]
    - “Exchanging, exchanging ... all the different experiences, points of view, the point of view of the doctor, the point of view of the nurse, the physiotherapist, the patient, the person...” [HP02: woman, 48 years, nurse, North]
    - “The achievement of the objective which, let us say, must be a multifactorial treatment. It's like we have problem with a nineteenth century machine and all its components have a defect, so we need an electrician, and a mechanic, etc. oh well. On this objective, the centers fail. They absolutely fail because they lack a vision, instead the patient migrates from one provider to another, and every provider has his own point of view, his own goals, and often the provider does not even listen to the patient.” [HP11: man, 45 years, physiotherapist, North]
    - “In fact... we do not have to arrive when there's nothing left to do, no. The work that must be done, is preparatory first. Otherwise it seems like… ‘the last beach’, when in reality it is not like that.” [HP10: woman, 42 years, psychologist, South]
    - “We need teambuilding. The team must be really connected, not only when you are hospitalized, not only inside the Center, but in general. What matters is that there is a real dialogue between the various players. That is absent, particularly between providers from different centers (silos working, lack of flexibility). We also need to dialogue with the patient and take into account the patient’s culture and capabilities.” [HP05: woman, 50 years, social worker, North]

- **Patient and SOs participation in multidisciplinary care (SO, Ns, HPs)**
  - SO:
    - "I participate in my relative's physio, also because not all the therapists know exactly the needs and problems of a given patient, we try to help having the most suitable and appropriate physio." [SO07: husband, 42 years, South]
  - Ns:
    - "Maybe the term 'SP' is important also to foster a greater involvement of the patient SOs." [N07: woman, 34 years, North]
    - "In teamwork, we should not forget the patient!" [N02: woman, 59 years, North]
    - "Patient involvement is vital as the knowledge of how my own body functions, how I can self-manage my own symptoms is an added (and necessary) resource for a multidisciplinary care. The patient must be on board, we have symptomatic treatments, some can function, but the future has the patient actively involved in his/her own care." [N01: man, 56 years, North]
  - HPs:
    - "The patient and his/her relative should become part of the equipe!" [HP05: woman, 50 years, social worker, North]
    - "We have to carefully listen to the patient. He/she is the real expert. He/she knows what is most important for him/herself." [HP03: woman, 56 years, physiotherapist, North]

- **A complete service charter of the MS center (HP)**
    - "All the features of the MS center should be presented to the patients already from the diagnosis, and not to say to the patient at some point, and out of the blue, 'It's time that you see a psychologist'. So, even in my center - and we're in a better situation compared to other - sometimes you see people hospitalized who did not know that there was the psychologist, they did not know... or at least they are referred when the problem is present from a long time, when it was possible to prevent it. Perhaps information should be given at the time of diagnosis, to take stock of the situation: 'Our center is organized like this, you can do this.' Then maybe you go to the psychologist in four years, but you know it was there." *[HP01: woman, 47 years, psychologist, North]*

- **Home visits/care (P)**
    - "Sometimes I am not able to come to the MS center, or it requires huge effort. It would be great if we could count on some specialist who can visit us at home!" [P03: man, 52 years, EDSS 6.0, North]

- **Fast and easy access to MS center and its professionals (P, SO, N, HP)**
  - P:
    - "What matters is always having the opportunity to have someone who, when you need, is here. The doctor, but also other HPs…" [P03: man, 52 years, EDSS 6.0, North]
  - SO:
    - "We need to have a facilitated access to the HPs. If we need to consult them, we need a way to reach them immediately." [SO04: wife, 49 years, North]
  - N:
    - "I think something really important for the patients and their families is to be able to contact us when they need it." [N01: man, 56 years, North]

- HP:
  - “Sometimes patients complain difficulty in having a quick access to the MS services. I think it would be great if they could easily access to it.” [HP05: woman, 50 years, social worker, North]

- **A case manager (Ps, SOs, HPs)**
  - Ps:
    - “A support, from specialized personnel, then from the hospital: a HP of a structure that would accompany me in such a delicate phase of my life.” [P01: man, 46 years, EDSS 6.5, North]
    - “Those people entering the tunnel of the disease with a higher level of problems would need a professional who coordinates the various disciplines... a project manager!” [P04: man, 44 years, EDSS 6.0, North]
  - SOs:
    - “A project manager is also needed.” [SO02: husband, 41 years, North]
    - “Ehm… we can say that in the last year we’re succeeding in having referral HPs a little more…” [SO06: daughter, 27 years, North]
  - HPs:
    - “I dream of a councelor at the center, who is a bit of a link. It occurred to me because we recently show a video with people with MS, and a lady with SPMS said, I'd like to have all these doors in front of me, with the various specialists, who give me advice. Then I find a person, that I call councelor, but call it what you want, who says to me: Well, you have these difficulties. And I'll direct you, I'll do a little connection to these specialists.” [HP05: woman, 50 years, social worker, North]
    - “If I were on the other side, on the side of the person with MS, I would need a reference center where I can call. Oh well, ok, I will be busy for two hours, in the end I will be able to talk to someone, and I think it would be of great support.” [HP02: woman, 48 years, nurse, North]

- **Rehabilitation: More physiotherapy/occupational therapy (Ps, SOs, Ns)**
  - Ps:
    - “From a practical point of view, some physio would help me, for sure.” [P05: woman, 44 years, EDSS 6.0, North]
    - “I always wonder why a center like XX does not have a service of physiotherapy.” [P06: woman, 39 years, EDSS 7.0, North]
  - SOs:
    - “I agree that home-based physio is good for… the soul, even in the case of my wife, who has gone from walking to the wheelchair in three days.” [SO02: husband, 41 years, North]
    - “The point is that they prescribe you physiotherapy, but just for a month.” [SO03: wife, 42 years, North]
    - “Then they withhold it to you.” [SO02: husband, 41 years, North]
    - “A month [physio] in a year, it is useless. I mean he does physiotherapy, but now it's daily. If you divide that month into one hour a day, that’s fine. But not a month in a year.” [SO03: wife, 42 years, North]
    - “Because that month there...” [SO02: husband, 41 years, North]
    - “That month is useful to you, but that's not enough.” [SO03: wife, 42 years, North]
    - “As you quit...” [SO02: husband, 41 years, North]

- "Then you come back as before." [SO03: wife, 42 years, North]
- "Come back... right." [SO02: husband, 41 years, North]
- "Because you can do the physio at home on your own, he does it every day at home ..." [SO03: wife, 42 years, North]
- "But it's not the same." [SO05: wife, 51 years, North]
- "What I miss most at this stage is the occupational therapist (OT)." [SO04: wife, 49 years, North]

- Ns:
  - "Very often, they have difficult situation. For example, it is hard to find the right place for rehabilitation. Parallel to this, we have no dedicated pathway to rehab and they have to wait 6 months at least!" [N05: woman, 50 years, South]
  - "The healthcare system provides them with only two rehab courses per year! However, these patients would benefit from a more continuous program. They ask us how they could do, but we have no answer!" [N07: woman, 34 years, North]

- **Collaboration with the general practitioner (HP)**
  - "It would be important to create a good collaboration with the general pratictioner. Now it is basically zero! It would be crucial because otherwise the patient comes to the MS center for everything!" [HP12: woman, 50 years, nurse, South]

- **Connection with AISM (SO)**
  - When a person receives the diagnosis, the MS center should create the connection with AISM. [SO04: wife, 49 years, North]

- **Psychological support for both patients and SOs (individual, couple, family or group setting; homogeneous (SPMS) group discussion for both patients and relatives (Ps, SOs, Ns HP)**
  - Ps:
    - "Having the opportuniy to speak with someone and share your feeling is really important. We need someone qualified, a psychologist!" [P06: woman, 39 years, EDSS 7.0, North]
    - "People should look for the help of a psychotherapist. I am doing a psychotherapy and this is helping me. I can clearly see how much I need it. Probably, I even needed to start before when I started!" [P04: man, 44 years, EDSS 6, North]
  - SOs:
    - "I think my husband really needs a psychological support! Unfortunately, this support is not available. My husband is so angry since the disease worsened that he really needs someone to talk to about his feelings." [SO05: wife, 51 years, North]
    - "Beside the physiotherapy, I think the role of the psychologist is crucial! The psychological support should be included and integrated in the activities of the center! I think it is even more important than the physiotherapy!" [SO01: wife, 48 years, North]
  - Ns:
    - "I think the psychological support should be available! It is so important!" [N01: man, 56 years, North]
    - "I agree, the MS center should provide a psychological support for both patients and their families." [N07: woman, 34 years, North]
  - HP:
    - "Transitioning to SPMS is a crucial step in this journey. It is like a second diagnosis. It requires a psychological support." [HP05: woman, 50 years, social worker, North]

- **Self-help groups (social networking; communication beyond self-help group e.g. MS café; "Happy hour for sharing" experience [only SOs permitted]) (P, SOs)**
  - P:
    - "You know what?! I really missed to share what I was living with other people that were dealing with the same difficulties." [P01: man, 46 years, EDSS 6.5, North]
  - SOs:
    - "Nobody can really understand if they are not living this situation. But being here and listening to her [another SO] story and her husband's reaction makes me feel I am not alone. At least I am not the only one!" [SO01: wife, 48 years, North]
    - Moreover, if we can share with other persons that are living the same situation we can also share information and suggestions! [SO01: wife, 48 years, North]

- **Psychological support for HPs (N):**
    - At the end, we've more challenges than the patients, perhaps then the care path should provide a support for the HPs, rather than just for the patient him/herself. [N05: woman, 50 years, South]

<u>Empowerment training</u>

- **Patients (i.e. nutritionist, how to deal with daily life, hints for searching information) (Ns)**
    - "You go along with the patient, helping him to have an appropriate lifestyle. You do it, you also talk about everything can help. This is becoming one of the... of the strategies, in short, working on the lifestyle, then on the sports. Then you can shift from sports to non-sport activities, to rehabilitation." [N02: woman, 59 years, North]
    - "Monthly meetings on specific topics, as we do in our center: as an example, we make a monthly meeting on putting nutrition into practice: we organize a snack in the afternoon in which we explain how to have breakfast at morning." [N02: woman, 59 years, North]
    - "I think that we should also organize meetings for patients on daily life management." [N04: woman, 50 years, Center]

- **HPs**

  Communication (Ps, N)
  - Ps:
    - "Surely knowing it, from an authoritative external source, would have given me a little more comfort, by being aware of the period I was going to live. Yes." [P01: man, 46 years, EDSS 6.5, North]
    - "I would advice the doctors to undress the gown and to immerge themselves a bit into the daily life of their patients... and therefore to try to be, as well as doctors, friends of their patients. Instead in the relationship doctor-patient, one is limited to describe the state of mind ... no ... not even the state of mind ... the physical state that a person has, labeling the person according to the the stage of the disease..." [P01: man, 46 years, EDSS 6.5, North]
    - "Maybe it's stupid what I'm about to say, but leave the person in his ignorance. Without insisting on giving information." [P05: woman, 44 years, EDSS 6.0, North]
  - N:
    - "We must improve our knowledge, know how to do it [communicate the transition] better. And we need tools that help us to communicate, definitely a crucial step. As for the communication of the MS diagnosis, where, thanks to the support of

different tools, we can give the patient a perspective: here [communication of SPMS transition] symptomatic therapies, rehabilitation, and... the capability to organize a new strategy of action." [N03: woman, 49 years, Center]

National and international cross training on SPMS (Meetings with multidisciplinary teams to learn how to deal with daily living activities; evidence based, up to date information about symptomatic treatments; lifestyle changes; disease modifying treatments) (SO, Ns, HPs)

- SO:
  - "[…] something that the XX could do, is a collaboration with... foreign hospitals. Some foreign hospitals have instruments that here in Italy are not on the list..." [SO02: husband, 41 years, North]
- Ns:
  - "Organise meetings to talk about some topics, inviting experts who can help both us and the patients." [N06: woman, 32 years, South]
  - "Train neurologists on these issues! We need to be well informed before providing information to the patient..." [N07: woman, 34 years, North]
  - "No, I think the training is valid for both doctors and patients. This is one of the key points... Doctors' training, as has already emerged, means that there is not only the neurologist's eye, and cross-training e.g. in MS rehab, via visiting periods etc (not just attending seminars) to learn how to deal with complexity at best, how to manage a chronic condition. We should find the resources for this kind of training, which is an investment. In fact, luckily our patients - differently from those suffering of cancer or ALS - they do not die. They are our patients for 40 years, which is an enormous burden. Here the resource????? is having the resources (i.e. the money). And relatives also need training, and information." [N01: man, 56 years, North]
- HPs:
  - "Training, training. That is, we need to train HPs so that they are able to act at their best. But if they are blockheads ... there is nothing to do." [HP02: woman, 48 years, nurse, North]
  - Many neurologists, even nurses, other HPs do not know how to do things. They pretend that the patient performs on his own (see e.g. self-cateterization). That is, it is easy to say "No, eh do not worry, there is the possibility that you do the catheterization". First, you should know the procedure, which is not easy, it's not that simple. And there is an emotional component as well. If you know what the procedure is, what the patient can do, you can also explain it to him properly and positively. [HP04: woman, 48 years, nurse, North]
  - "Training of the team, the team that needs networking, but not only in the hospital setting." [HP05: woman, 50 years, social worker, North]
  - This experiential training can really make a difference for the HPs [...]. It still happens that even young girls hospitalized in rehab dept, during the day they take their rehabilitation sessions, then at night the nurse says "I'll put on your night diaper…" [HP01: woman, 47 years, psychologist, North]

SOs (i.e. disease and patient management; how to deal with daily life; how to deal with children in case one of the parents has SPMS) (P, SOs, Ns)

- P:
  - “I think it is very useful, first of all to be prepared for what can be the change, the change of life of the person you have next, and then to become aware of what actually is the problem…” [P01: man, 46 years, EDSS 6.5, North]
- SOs:
  - “Then I said, Did I do the right thing? Was I wrong? Involve a girl [the daughter] in this thing? Or will it make her... ... a better person? I do not know. These things ... I miss a little this approach ... that is, a [name of the neurologist] for the physio, a [name of the neurologist] for ... that is, no?” [SO04: wife, 49 years, North]
  - “I would like someone to do me a course, to say: Because I think 100% I do not even know what's going on. Sometimes I become aggressive, unintentionally I do humiliate him. The point is that I would like to know how to handle this situation in terms of OT, to understand also what to do inside my house. Sometimes I also have a tendency to run. Like the other day, I was on the internet looking for lifters of all kinds, right? But maybe he, for now, does not need it at all, and maybe I'm humiliating him, talking about lifters to him - a man who is 50, has post-graduation, a work, speaks 5 languages, is an intelligent man. But I'm talking about the lift on the toilet, right? Maybe sometimes I even forget to have a daughter at home, I talk to her about these things.… I deserve help too, as I’m the caregiver! All in all, I’m the second impacted person because of the disease. If I think to my mother-in-love….she’s awful!” [SO04: wife, 49 years, North]
  - “There is absolutely no instruction manual. But on one thing I agree with what you said, that we need instructions for us. There is no instruction manual, absolutely. I give you an example: they prescribed the wheelchair, choosing it without considering my wife needs, and now we are having the changes to the chair on our own pocket. Another example: getting on the car. A trivial procedure for every person, which is done every day, twenty times a day. We do not notice it. With my wife it is the hell. Because you do not know exactly how to take her, how to put her in, how to turn her, then she gets angry… in short, it is the slaughter that we all know.” [SO02: husband, 41 years, North]
- Ns:
  - “Monthly meetings also in groups, even with family members, because of course the support of family members, e.g. in the case of nutrition is not so trivial.” [N02: woman, 59 years, North]
  - “An active part, let's say, a small slice of the cake must also be dedicated to the family, to educate it, instruct the family on nutrition, on the importance of certain things...” [N07: woman, 34 years, North]

<u>Information</u>

- **Information on SPMS for patients and SOs (Ps, SOs)**
  - Ps:
    - “A kind of booklet… the disease has changed, maybe I need psychological support, where can I go? Try to provide tracks that one can walk.” [P06: woman, 39 years, EDSS 7.0, North]
    - “Yes, because often knowledge makes, say, you strong. And if you are not afraid of the future, the bulk of the work is already done. To be aware...” [P01: man, 46 years, EDSS 6.5, North]

- SOs:
  - “Every time I go to the doctor, the last question I ask him is this: What is waiting for us?" [SO04: wife, 49 years, North]
  - “I missed someone who said me: "I'm sorry to say it, but if there's no news from the MRI, it's not even a good thing, because it means he's in the progressive phase. He no longer has relapses, because everything has become more difficult, because the disease is now progressive. I mean, I wanted to know a little bit of everything.” [SO04: wife, 49 years, North]
  - “Information must be given. It should also be given plenty (kilos) of information, not just to the caregiver, but also to the children. They all must be aware of everything, time by time.” [SO07: husband, 42 years, South]

- **Clear information on non-pharmacological approaches and treatment/lifestyle/aids (P, SOs, N, HPs)**
  - P:
    - “I would find very useful to inform about the different disease forms and what to do to adjust to each form. It should help a lot…” [P01: man, 46 years, EDSS 6.5, North]
  - SOs:
    - “What I’m missing, as also pointed out by the other participant, is a... another type of catalogue, let's say. A catalogue with a compendium of all possible and imaginable aids. We recently discovered a... a wheelchair with BMX wheels, double, to go to the beach.” [SO07: husband, 42 years, South]
    - “Even the electrical stimulator, we discovered it by accident, having a tea in the afternoon with this guy, known by chance. All by chance. No? We discovered the scooter because I was in Northampton and I saw them around. And I said’this is a fantastic stuff.’ So I started asking colleagues...” [SO02: husband, 41 years, North]
  - N:
    - “There are so many aids, and we in our offices are worried as we are competent in monitoring drugs, we do not even know what these aids are, but maybe... collaborating with the physiatrist...” [N04: woman, 50 years, Center]
  - HPs:
    - “It should start from the neurologist the idea that we’ve not a drug to cure the disease but we have rehab, and a treadmill is needed, and we provide advice on the treadmill...” [HP05: woman, 50 years, social worker, North]
    - “Yes, diet, healthy habits, alternative therapies, [...] It would be a way to take care of the patient who really with the SPMS has a bit of abandonment syndrome, because he no longer has those scheduled appointments, he feels lost.” [HP05: woman, 50 years, social worker, North]
    - “In this phase the physiotherapist, by working with the patient, with the person, has the right approach to try also those aids which the patient has refused, sometimes for so long…” [HP02: woman, 48 years, nurse, North]
    - “To accept aids. To accept...” [HP04: woman, 48 years, nurse, North]
    - The patient came in, he said, "I'm not thinking that I'm going to walk again, it's not that. But I would like something to stand up, to reinforce the trunk anyway." But that patient was not receiving anything of the kind, as if he had been given for... [HP03: woman, 56 years, physiotherapist, North]

- **Social care information (i.e. Italian law 104) (SO, N)**

- SO:
  - “I discovered the law 104 only two years ago, while we could have benefited from it since long. Nobody informed us about this before, I knew it from the labor unions.” [SO01: wife, 48 years, North]

- N:
  - “We cannot even keep up of all the social aspects. See e.g. the recognition of disability, the first appeal, the second appeal, the certificate is wrong, the 104 law was not recognized. This all increases the frustration and leads the patient into a vicious circle, because after him he expects these benefits, and these benefits are not received, instead of motivating him we demotivate him even more. And on the other side, there is a job for the neurologist that becomes stratospheric...” [N05: woman, 50 years, South]

- **Website (or other sources) to share solutions and information (P, SO)**
  - P:
    - I spend my time saying, "But will this exist?" And I go in the internet, and sometimes I say despite I studied English I can not find things. But I realize that it is really a, not a trivial expenditure. Sometimes I think: "It's not that all people maybe want to do these things." and sometimes I think I should pick all the stuff I found on a website, but since my technological capacity is zero... "Wow, but maybe it would be useful, for other people like me, to have ..." And so I would like to do a kind of booklet... [P06: woman, 39 years, EDSS 7.0, North]
  - SO:
    - “A blog! A site in which all describe the solutions they found to their problems, so that you do not need to reinvent the wheel…” [SO04: wife, 49 years, North]

<u>Polices</u>

- **Job outplacement (P, SOs)**
  - P:
    - “To scale back, also at work...finding a suitable job, a part time job, that can be reconcilied with my care pathway, the physio… Today, as a commuter worker, it’s impossible to me to carry out other activities because I arrive so late in the evening that for me the day is already over...” [P01: man, 46 years, EDSS 6.5, North]
  - SOs:
    - In the end, eh ... he got along with his boss, who said: "Don’t worry. Stay at home, take care of yourself, take your treatments…” but they fired him! And after that he got worse, just saying: "Now how ...? What can I do?” [SO05: wife, 51 years, North]
    - They should help a disabled person. To say, my husband works in XX [a town in the hinterland of Milan], and we live here behind [downtown Milan]. He used to go to work by public transport, but now he cannot do it anymore. He drives. The regional health system should help my husband to find a job near home. You cannot expect a disabled person to travel miles to get to work... Just support him so that he can keep his job! Because if I had him at home... But I prefer that he keeps going! I know how much it costs to him, but he is busy. In those hours at work he is occupied, his brain works, he has a commitment. And it's something important... to have a purpose. He says, "I have to gain the family income." In fact I don’t work, and the scope of my husband is to provide the income for our family. If he stopped working… Rather, it

would be great if [the system] could help people like my husband to retain their job, even a part-time one, but near home. [SO03: wife, 42 years, North]

- **Improving the social security net (P, HP)**
  - P:
    - In my opinion, in Italy, we are really in a bad situation as we lack the sense of "welfare". Last summer I went to England, and going around the city was not a problem: there is the disabled service you call and, if you book early, take you where you want in the city. You need, let's say the shopping at home? You call them, they bring the shopping to you. Hmm... I wanted to go to London alone by train. I said, "Ok, now you've done 30, you do 31." This is what we really miss: services at a social level that make everything easy. [P06: woman, 39 years, EDSS 7, North]
  - HP:
    - "An assistance network in the area." [HP10: woman, 42 years, psychologist, South]

- **Facilitate bureaucracy (P, SO)**
  - P:
    - "All this bureaucracy is killing us." [P04: man, 44 years, EDSS 6.0, North]
  - SO:
    - […] simply because they can't leave their house! I had to make a change to my house, and in the end I did it without asking permission, and paid it all. Why? Because when I went to the municipality to ask for... what should be quite trivial, having a ramp. We live on the ground floor, I had to make the ramp to allow... to enter from the balcony and go directly into the kitchen, because to get into the house we have the steps, from the main entrance. And they said: "Well, prepare these things, and in six or eight months you will have the permission." And I said: "Guy, in these months how can I bring my wife home?" I mean, I carried her in my arms… Though I'm lucky, compared to my colleagues who are here, I'm lucky that my wife weighs 50 kilos ... [SO02: husband, 41 years, North]

- **Supporting patient's autonomy/accessibility (P, SO, HPs)**
  - P:
    - This is what I'm missing. Resources that on a social level that make everything simpler. And I'm not reasoning about big issues, but just concrete, small issues that make a difference. If I should say "I go to Milan alone, from my house", I can't do it, because I should find a bus that takes me to the railway station, and the buses are not accessible; and then I should… [P06: woman, 39 years, EDSS 7.0, North]
  - SO:
    - Eh. We are at the Stone Age, because when we go to London, when we go to Copenhagen and so on, you, believe me, in London I went to see the football game, my wife went with my daughter to go shopping. And I did not worry about anything. Because I know they were back safely, with bags full, happy and happy with the day. Here I do not trust to leave them for two minutes. Because that's how it is. Because here, going to have breakfast is already a hell, as you can't go to the bar. All the bars, no exception! In England, we were in a village ... no more than 40 houses and a pub. And while I was looking for a parking machine nearby, my wife said: "I'm going to look at the bar." She went ahead with our daughter. Then I called her on the phone and said "Where are you?" And while I was approaching the bar, they opened

the door, they accompanied me, they made me sit down, and in a while I found myself seated at the bar, ordering!" [SO02: husband, 41 years, North]
I mean, it's a relationship ... In other countries it’s different.
Facilitator: Autonomy is enhanced.
Exactly [SO02: husband, 41 years, North]
Facilitator: Makes autonomy possible.
In fact, we are now thinking of buying a house in England, I’d like to stay there three months a year, working from remote... and she can walk around quietly. Because we, in our country, in a village, there, we can’t go, she can’t go around alone. Why? Because there... the sidewalk ends up with the step. There are no ramps. It’s trivial, but these issues are not considered at all. [SO02: husband, 41 years, North]

- HPs:
    - “We should keep focused in improving something in, in their daily life ... And I'm interested in just that they do it in the neighborhood, that they get off more, that they can walk more. That’s very important.” [HP03: woman, 56 years, physiotherapist, North]
    - “Instead it falls into a cauldron, we do not have time, as an example instead of supporting you in your daily activitities, we prefer to offer another solution, as just do it [urinate] in the bed. It is not right! What about the person’s dignity? That is, you have to stay with him to accompany him in the bathroom, to instruct him how to do it autonomously, caring for him and supporting him.” [HP02: woman, 48 years, nurse, North]
    - “The patient should acquire the skills to have the most participatory life that is possible. Within this... curriculum, the focus is not the cure, but it is living well, and the resources are not clinical, but they are, precisely, peer support, family support, etc. This requires a specialized and multi-disciplinary intervention. Here the goal of care is the preservation of social participation.” [HP11: man, 45 years, physiotherapist, North]

- **Developing specific national guidelines for people that are experiencing SPMS transition (HP)**
    - “To set up guidelines.” [HP10: woman, 42 years, psychologist, South]
