# Supplementary material for: Conversion to secondary progressive multiple sclerosis: Multistakeholder experiences and needs in Italy
Source: PLoS One. 2020 Feb 13;15(2):e0228587. doi: 10.1371/journal.pone.0228587 (PMC7018010; doi:10.1371/journal.pone.0228587)
Supplement: Attachment — Submitted filename: Replies to Reviewers.docx [file pone.0228587.s004.docx]

**Responses to the reviewers**

**Reviewer #1**

1. *I do not understand why in the introduction authors mention also the German chapter of this research, but, then, did not report on this. Are there any methodological differences in study conduction between Germany and Italy justifying two different papers based on the same project? Otherwise, I do expect to get presented both. It would be very much helpful also comparing Germany and Italy to have better insight into this issue.* We thank the reviewer for his comment. As from the published protocol (manuscript’s reference 12), the ManTra project originated in Italy and it was then adopted in Germany. Concerning the qualitative study (ManTra project action 2), the eligibility criteria and the interview guides were the same in the two countries. However, for feasibility reasons, the German protocol differed in the following: a) patient personal semistructured interviews (PSIs) were replaced by telephone PSIs in Northern Germany, and by a focus group meeting (FGM) in Southern Germany; b) patient significant other PSIs were replaced by a FGM; c) Instead of two FGMs (one with neurologists, and one with other health professionals) there was one FGM with neurologists and other health professionals. We agreed to present the findings of this project action separately in view of these differences, and also because the analysis was run separately in the two countries by Italian and German researchers in order not to miss any conceptual and semantic nuance. Reporting all the process in a single manuscript would have been too much, even in a journal that has not space constraints. In the following manuscript, we will describe the German qualitative study, consider contrasts and similarities of findings across the two countries, and highlight the need of a guidance document for qualitative analyses of data from different languages/cultures.
2. *The paragraph “In line with the above studies… before SPMS was medically confirmed” should be moved from the Introduction to methods and results, as appropriate.* *Also, I would move the paragraph “Managing the transition to SPMS… newly diagnosed patients with SPMS” at the very end of the introduction, since it contains study objectives.* The manuscript reports the results of one action (action 2, Italian findings) of the ManTra project. For this reason, in the Introduction we included a description of the project at large. However, the present point and the following one indicate that we were not clear enough in our description. Thus, following the reviewer’s advice we have re-shaped the Introduction by outlining more briefly the ManTra project, at the end of the Introduction, specifying in the last sentence the objective of the present paper.
3. *A key point of this manuscript is the lack of awareness of patients and the variability of neurologists’ attitude towards SP conversion. As a matter of fact, a diagnosis of SPMS limits the possibility of DMT prescription and, more in general, of room for improvement in MS management. These could be responsible at least in part for the delay in officially identifying SPMS in neurology clinical practice.* We agree with the reviewer that the limited availability of DMT for SPMS is one reason for postponing the disclosure of SP conversion (see e.g. Discussion: ‘In particular, neurologists may be reluctant to recognize the bad prognosis and the scarcity of effective disease-modifying treatments for this disease form. As a result, they may avoid having conversations on these topics with patients and their families.’).
4. *In the limitations’ section of the discussion, when discussing on the SOs sample, I would suggest authors refer to Neate et al Plos One 2019.* The paper by Neate et al. is an interesting one on the experiences of partners of people with MS who attended a healthy lifestyle workshop. Nevertheless, this study does not refer to our difficulty in enrolling significant others (SOs) from the South of Italy, and SOs of SPMS patients with severe cognitive compromise.
5. *Also, SP patients suffer from cognitive impairment at some level (Moccia et al. Mult Scler 2016), and formal testing would have been helpful.* We agree that cognitive compromise is common in SPMS. To safeguard the validity of our findings, patients with severe cognitive compromise (referring neurologist judgment) or unable to communicate effectively were excluded. We purposely avoided to exclude patients with mild or moderate cognitive impairment, and deemed that a formal cognitive testing was not necessary. Notably, we did not include patients whose SPMS conversion dated over the preceding 5 years.

**Reviewer #2**

1. *Since lack of SPMS awareness in patients resulted as one of the major findings it would be interesting to understand whether aware and unaware MS patients showed any relevant difference in terms of the two themes "Dealing with symptoms worsening" and "Needs".* Participants who were unaware of the transition were less informative and vaguer in their narratives. Therefore, we cannot be sure that differences in the categories reported by the two subgroups are due to different experiences or are simply the results of the difficulties of the unaware patients in speaking about the topic. Moreover, we cannot guarantee that the saturation criterion is respected if we run separate analysis for aware/unaware patients.

On pages 13-14 below we have re-organized the two themes "Dealing with symptoms worsening" and "Needs" based on patient awareness. Taking into consideration the limitations reported above these results are only exploratory, and we prefer not to include them in the manuscript. Common subcategories for unaware and aware patients are reported in red.

2. *Authors should explain more in detail study limitations regarding low sample size - particularly in subgroups - and lack of a standardized definition of SPMS among other factors.* In the Discussion, we have acknowledged among the study limitations the restricted number of SOs from the South of Italy, and the lack of SOs of patients with severe cognitive compromise. Nevertheless, any subgroup analysis is out of the scope of qualitative analysis. Regarding the SPMS criteria, we used Lublin 1996 (reference 2 of the manuscript).

**Theme 3 – Dealing with symptom+MS worsening**

| ***Categories*** | ***Personal Experience*** | | ***Adjustment strategies*** | | ***Difficulties in adjustment*** | |
| --- | --- | --- | --- | --- | --- | --- |
|  | *Unaware* | *Aware* | *Unaware* | *Aware* | *Unaware* | *Aware* |
| Sub-categories | - Guilt *(Ps, SOs)* - Sadness *(Ps)* | - Anxiety *(Ps)* - Anger *(Ps, SOs)* - Fear *(P, SOs, HPs)* - Sadness *(Ps)* - Loneliness of both patients and SOs *(Ps, SOs)* - Humiliation/shame *(Ps, HPs)* - Disappointment *(Ps, SOs)* | *Concrete solutions*   - Autonomy maintenance *(Ps, SOs)* - Work maintenance *(P, SOs)* - Staying in contact with the neurologist or HPs *(Ps, SOs, Ns, HPs)*   *Inner resources*   - Tenaciousness *(Ps,)* - Acceptance *(Ps)* | *Concrete solutions*   - Surfing the web *(Ps, SOs)* - Looking for new drugs *(Ps, SOs)* - Autonomy maintenance *(Ps, SOs)* - Work maintenance *(P, SOs)* - Planning activities carefully (one activity at time) *(Ps)* - Self-care *(Ps)* - Staying in contact with the neurologist or HPs *(Ps, SOs, Ns, HPs)*   *Inner resources*   - Grounding *(Ps)* - Acceptance *(Ps)* - Meaning *(Ps, SOs)*   *Spirituality*   - Religion/faith *(Ps)*   *Social connectedness*  Social support *(Ps)* | *Inner factors*   - Experiential avoidance/denial *(Ps, SOs)* | *Inner factors*   - Giving up *(Ps, HPs)* - Not sharing *(Ps)* - Retirement from the relationship *(Ps)* - Cognitive fusion *(Ps)* - Experiential avoidance/denial *(Ps, SOs)*   *Environmental factors*   - Uncertainty *(Ps, SOs)* |

**Category 1: Personal experience**

Unaware

- **Guilt**
  - I have tried to take care of me, but then I felt guilty for all the things I failure… It’s my fault! I could do more… [P05: woman, 44 years, EDSS 6, North]

Aware

- **Anxiety**
  - Oh yes, I felt so anxious! [P06: woman, 39 years, EDSS 7, North]
- **Anger**
  - I get angry. Just an example, I need a pen, so I say, "Ok now I stand up and go. Now I stand up and go.” However, at the end, I can’t stand up and I can’t go! So I have to ask someone, and I start feeling so angry! [P06: woman, 39 years, EDSS 7, North]
- **Fear**
  - I was totally afraid! [P15: man, 39 years, EDSS 7, South]
  - What I am experiencing is pure fear! It is a deep fear! How can I go to work? How can I reach a certain place? It is a fear about how to do practical things. [P04: man, 44 years, EDSS 6, North]
  - I am afraid of the future, especially when the future is like a mountain, absolutely bigger than you! [P01: man, 46 years, EDSS 6.5, North]
  - I have this feeling that if I spend too much time seated I will not walk anymore! [P06: woman, 39 years, EDSS 7, North]
- **Loneliness of both patient and SOs**
  - I have spent many years without telling anybody about it, without saying it to myself. [P13: woman, 42 years, EDSS 6.5, South]
  - You feel alone. [P11: man, 47 years, EDSS 6.5, Centre]
  - The result was that I have managed all this worsening all alone. [P01: man, 46 years, EDSS 6.5, North]
- **Humiliation/Shame**
  - The most humiliating aspect of the disease is to not be able to take care of my son, to play with him, supporting him while is growing up. I cannot play with him as the other dad does. I cannot give a horseback ride to my son. My wife has a huge burden. I have to step aside very often, this is humiliating! [P01: man, 46 years, EDSS 6.5, North]
  - I was ashamed! [P13: woman, 42 years, EDSS 6.5, South]
- **Disappointment**
  - It is a pity that even the symptomatic drugs have no effect on me. It is really disappointing! [P01: man, 46 years, EDSS 6.5, North]

Both

- **Sadness**
  - I lost my autonomy, this makes me really sad! [P15: man, 39 years, EDSS 7, South]
  - Right now, speaking about it, a drop falls down on my cheek. [P05: woman, 44 years, EDSS 6, North]

**Category 2: Adjustment strategies**

Unaware

Inner resources

- **Tenaciousness**
  - You have to keep going no matter what! Whatever is going to do, you need to try [P02: woman, 60 years, EDSS 4.0, North]

Aware

Concrete solutions

- **Surfing the web**
  - I usually surf the web, read the forums, I do my best to keep me informed about new drugs… [P11: man, 47 years, EDSS 6.5, Center]
  - I was a little confused… I didn’t know… I started to look for information on the internet in order to see if I could find a possible, alternative cure. [P01: man, 46 years, EDSS 6.5, North]
- **Looking for new drugs**
  - We are on hold […] waiting for the Doctor to say: “Massimo, there is this new drug” or “Massimo, we are studying…” for example we are waiting for the stem cells. We know that it will take long… three years at least!” [P11: man, 47 years, EDSS 6.5, Center]
  - I often ask if new drugs are available. [P01: man, 46 years, EDSS 6.5, North]
- **Planning activities carefully (one activity at time)**
  - I learned to focus on one activity! For example, I could say: “today I cook everything by myself!” It becomes my main activity that day! [P06: woman, 39 years, EDSS 7.0, North]
- **Self-care activities (P):**
  - I think every person needs to dedicate time to him/herself! Such moments make you feel a sense of achievement. I find this in religion and going to the gym. These two commitments help me hugely! [P01: man, 46 years, EDSS 6.5, North]

Inner resources

- **Grounding**
  - I felt like in the middle of a storm, you try to rely on something that can support you, an anchor for the future. While dealing with my “physical storm” I looked for some pillars that supported me, such as my job, my faith and my son. These were my three elements.” [P01: man, 46 years, EDSS 6.5, North]
- **Meaning (Ps, SOs)**
  - Patients’ quote:
    - “The only positive thing in this moment is that you realize what really means to you in life! What are the really important things”. [P01: man, 46 years, EDSS 6.5, North]

Spirituality

- **Religion/faith (P)**
  - Patients’ quote:
    - I have found some relief in religion; I always had a good relationship with God. The church has been my only outlet. [P01: man, 46 years, EDSS 6.5, North]

Social connectedness

- **Social support (P)**
  - Patients’ quote:
    - Having many friends helped me a lot! Telling them about my state and emotions helped me a lot! [P06: woman, 39 years, EDSS 7.0, North]

Both

Concrete solutions

- **Autonomy maintenance**
  - “If my husband is away on a business trip, my mom stays with me and sleeps with me. In this way, I am not alone if my daughter needs something or I have to weak up during the night. Of course, my mom can’t pick up me and I can keep working on my autonomy, even when she stays at our place. [P06: woman, 39 years, EDSS 7.0, North]
  - When I prepare the meal I can be a little unbalanced. No worries, I put all the ingredients on the table, I sit down and keep going … same thing with ironing.” [P02: woman, 60 years, EDSS 4.0, North]
- **Work maintenance**
  - I have always tried to keep my job, to never leave it, because it is a very important aspect of my life. My job is my anchor to the reality, to the person I was before. [P01: man, 46 years, EDSS 6.5, North]
  - My job helped me in avoiding desperation and moping. I found keep working really useful! [P02: woman, 60 years, EDSS 4.0, North]
  - Sometimes I really want to react to these difficulties and looking for a way to overcome them! My job is a key factor! I have always tried to keep my job and never give up on it. My job anchors me to the reality; to the man I used to be! [P01: man, 46 years, EDSS 6.5, North]
  - I have a part-time work, 9 hours per week, but I have decided to increase the amount of hours to 12 the next year. Instead of laying without nothing to do, it is so much more meaningful to work and being with other people. [P06: woman, 39 years, EDSS 7.0, North]
- **Staying in contact with the Neurologist or HPs**
  - Above all, the most important thing is that if I had a doubt I can write to the neurologist and receive an answer. [P06: woman, 39 years, EDSS 7, North]
  - I am under Dr. XX’s care. He is always available, this is fundamental! To be able to rely on someone when you need that, it’s crucial! It is not in terms of physical presence, sometimes there is nothing practical he can do, but to know there is someone you can rely on… someone with which you can discuss your situation, this is the most precious thing. [P07: man, 49 years, EDSS 6, North]

Psychological skills

- **Acceptance (Ps)**
  - It is important to recognize that it is not “the end of the world” and accept the limits I have. [P06: woman, 39 years, EDSS 7, North]
  - It has been a while since I started perceiving a feeling of resignation. Something like “this is my life, even though my projects for the future were different”. It is what it is, hence I am trying to live my life in the best way possible. [P15: man, 39 years, EDSS 7, South]
  - Acceptance is really important. It implies a sense of proactiveness. Acceptance means being aware of the situation and it is crucial to be effective in taking any decision. What can I do if I need to go from A to B and I am encountering some barriers? I can be stuck somewhere, or I can accept it and seeking a way to overcome it, maybe walking around the “stone”, going back and taking another road, finding other strategies. I know I have the tendency to avoid thing but, in the end, you have to deal with what is going on. [P07: man, 49 years, EDSS 6, North]

**Category 3: Difficulties in adjustment**

Unaware

Inner factors

- **Experiential avoidance/denial**
  - Well, it is like I said “This is better, I have not to take any drugs… I don’t want to think about the disease”. I still avoid thinking about this problem. This is true also with other people, I am doing my best to hide it to others. [P12:woman, 56 years, EDSS 6.5, South]

Aware

Inner factors

- **Giving up**
  - I just want to leave everything, go in a hospital, and stay quiet there. [P01: man, 46 years, EDSS 6.5, North]
  - The most important thing in life is don’t put yourself down. I fell and got up so many times… you really need to fight in life. [P03: man, 52 years, EDSS 6.0, North]
  - I have this feeling of resignation… even though my projects were different, this is the life I have. [P15: man, 39 years, EDSS 7.0, South]:
  - The elderly man usually says: I know it would have been this way. What else can I do? [HP04: woman, 48 years, nurse, North]
- **Not sharing**
  - I’d rather to do it myself. Others wouldn’t understand. [P10: man, 41 years, EDSS 4.5, Center]
  - I didn’t share it [the transition to SMSP] with almost anybody. I didn’t have the opportunity to speak about it with anybody, neither with doctors nor relatives. [P01: man, 46 years, EDSS 6.5, North]
- **Retirement from the relationship**
  - I withdrew from the world. My life is now the PC, the TV and playing with crosswords. Sometimes my daughter visits me but it is no more the same. Also about meeting other people, it is so rare! [P03: man, 52 years, EDSS 6.0, North]
- **Cognitive fusion**
  - I have this feeling that if I spend too much time on the wheelchair, I wouldn’t never walk anymore! Therefore, if I have to do something, and I need the wheelchair to move, I keep having this thought and I remain stuck! It is a nonsense because I simply threw away two hours thinking about it when I could do this in a couple of minutes! [P06: woman, 39 years, EDSS 7.0, North]

Environmental factors

- **Uncertainty**
  - It would have been of help to be more informed and aware of what was going on. It would have helped me in not being alone in this process. I wondered “Will it be in this way forever? Will it be worse? What I am going towards in my life? I really would appreciate to receive more information! [P01: man, 46 years, EDSS 6.5, North]

**Theme 4: Needs**

| ***Categories*** | ***Met needs*** | | ***Unmet needs*** |  |
| --- | --- | --- | --- | --- |
|  | *Unaware* | *Aware* | *Unaware* | *Aware* |
| Sub-categories | *Organization and management*   - Neurologist availability *(Ps, SOs, Ns)* - Physiotherapy *(Ps, SOs, Ns, HPs)* | *Organization and management*   - Neurologist availability *(Ps, SOs, Ns)* - Physiotherapy *(Ps, SOs, Ns, HPs)* - Connection with Italian MS Society *(Ps, SOs, Ns, HPs)*   *Information*   - Easy access to information about aids *(Ps, SOs)* | *Organization and management*   - Rehabilitation: More physiotherapy/occupational therapy *(P, SOs, Ns)*   *Empowerment Training*   - HPs - Communication *(Ps, Ns)* | *Organization and management*   - Ad hoc plan of follow up visits with the neurologists and other HPs *(Ps, Ns, HPs)* - Multidisciplinary care/holistic approach *(Ps, SOs, Ns, HPs)* - Home visits/care *(Ps)* - Fast and easy access to MS center and its professionals *(Ps, SOs, Ns, HPs)* - A case manager *(Ps, SOs, HPs)* - Rehabilitation: More physiotherapy/occupational therapy *(P, SOs, Ns)* - Psychological support for both patients and SOs (individual, couple, family or group setting; homogeneous (SPMS) group discussion for both patients and relatives *(Ps, SOs, Ns, HPs)* - Self-help group (social networking; communication beyond self-help group e.g. MS café; “Happy hour for sharing” experience (only SOs permitted) *(Ps, SOs)*   *Empowerment Training*   - HPs - Communication *(Ps, Ns)* - SOs (i.e. disease and patient management; how to deal with daily life; how to deal with children in case one of the parents has SPMS) *(Ps, SOs, Ns)*   *Information*   - Information on SPMS for patients and SOs *(Ps, SOs)* - Clear information on non-pharmacological approaches and treatment/lifestyle/aids *(Ps, SOs, Ns, HPs)* - Website (or other sources) to share solutions and information *(Ps, SOs)*   *Policies*   - Job outplacement *(Ps, SOs)* - Improving the social security net *(Ps, HPs)* |

**Category 1: Met needs**

Unaware

Aware

Organization and management

- **Connection with the Italian MS Society, AISM**
  - AISM has been a great discovery! It is not only about fundraising, they really help you! [P04: man, 44 years, EDSS 6.0, North]

Information

- **Easy access to information about aids**
  - Every time I can’t do something I look for some aids, tools or strategies that let me do the things I did before! [P06: woman, 39 years, EDSS 7.0, North]

Both

Organization and management

- **Neurologist availability**
  - It is particularly important to me that, whenever I need, I can write to my neurologist and receive a reply!” [P06: woman, 39 years, EDSS 7.0, North]
  - I really sing the praises of my neurologist! He is always available! This is really crucial, I can always count on him if I need him! Even if he cannot solve the problem, because there is not solution, to know that he’s available and willing to help is a big thing! You can always count on him! [P07: man, 49 years, EDSS .06, North]
  - It has been very helpful to me to come here and speak with the doctor: it is a real outlet for me. Maybe it doesn’t change the reality, because there is nothing you can do, but it makes you feel cared for! [P02: woman, 60 years, EDSS 4.0, North]
- **Physiotherapy**
  - Physiotherapy is crucial! The intensive rehabilitation is always something that helps in dealing with new difficulties and limitations you encounter during disease progression. Every time I went there I found it really beneficial at both physical and mental level.” [P07: man, 49 years, EDSS 6.0, North]

**Category 2: Unmet needs/resources**

Unaware

Aware

Organization and management

- **Ad hoc plan of follow up visits with the neurologists and other HPs (i.e.** diagnostic therapeutic care pathway, PDTA**)**
  - Yes, in my opinion, it's an important thing. Because if I have to have a consultation and it is only possible after two months, at that time the consultation can be useless (e.g. the symptom has disappeared) [P03: man, 52 years, EDSS 6.0, North]
- **Multidisciplinary care/holistic approach**
  - …and you know that the physiotherapist or the physiatrist can talk to your neurologist. That is, the neurologist, the psychologist, the psychotherapist, the physiatrist, the physiotherapist ... talk to each other about you, using a scientific and rational language, different from the lay language of patients. [P06: woman, 39 years, EDSS 7.0, North]
- **Home visits/care**
  - Sometimes I am not able to come to the MS center, or it requires huge effort. It would be great if we could count on some specialist who can visit us at home! [P03: man, 52 years, EDSS 6.0, North]
- **MS center on call**
  - What matters is always having the opportunity to have someone who, when you need, is here. The doctor, but also other HPs… [P03: man, 52 years, EDSS 6.0, North]
- **Case manager**
  - A support, from specialized personnel, then from the hospital: a HP of a structure that would accompany me in such a delicate phase of my life. [P01: man, 46 years, EDSS 6.5, North]
  - Those people entering the tunnel of the disease with a higher level of problems would need a professional who coordinates the various disciplines ... a project manager! [P04: man, 44 years, EDSS 6.0, North]
- **Psychological support for both patients and SOs (individual, couple, family or group setting; homogeneous (SPMS) group discussion for both patients and relatives**
  - Having the opportunity to speak with someone and share your feeling is really important. We need someone qualified, a psychologist! [P06: woman, 39 years, EDSS 7.0, North]
  - People should look for the help of a psychotherapist. I am doing a psychotherapy and this is helping me. I can clearly see how much I need it. Probably, I even needed to start before when I started! [P04: man, 44 years, EDSS 6, North]
- **Self-help groups (social networking; communication beyond self-help group e.g. MS café; “Happy hour for sharing” experience (only SOs permitted))**
  - You know what?! I really missed to share what I was living with other people that were dealing with the same difficulties. [P01: man, 46 years, EDSS 6.5, North]

Empowerment training

- **SOs (i.e. disease and patient management; how to deal with daily life; how to deal with children in case one of the parents has SPMS)**
  - I think it is very useful, first of all to be prepared for what can be the change, the change of life of the person you have next, and then to become aware of what actually is the problem… [P01: man, 46 years, EDSS 6.5, North]

Information

- **Information on SPMS for patients and SOs**
  - A kind of booklet… the disease has changed, maybe I need psychological support, where can I go? Try to provide tracks that one can walk. [P06: woman, 39 years, EDSS 7.0, North]
  - Yes, because often knowledge makes, say, you strong. And if you are not afraid of the future, the bulk of the work is already done. To be aware ... [P01: man, 46 years, EDSS 6.5, North]
- **Clear information on non-pharmacological approaches and treatment/lifestyle/Aids**
  - I would find very useful to inform about the different disease forms and what to do to adjust to each form. It should help a lot… [P01: man, 46 years, EDSS 6.5, North]
- **Website (or other sources) to share solutions and information**
  - I spend my time saying, "But will this exist?" And I go in the internet, and sometimes I say despite I studied English I cannot find things. But I realize that it is really a, not a trivial expenditure. Sometimes I think: "It's not that all people maybe want to do these things." and sometimes I think I should pick all the stuff I found on a website, but since my technological capacity is zero... "Wow, but maybe it would be useful, for other people like me, to have ..." And so I would like to do a kind of booklet... [P06: woman, 39 years, EDSS 7.0, North]

Polices

- **Job outplacement**
  - To scale back, also at work ...finding a suitable job, a part time job, that can be reconciled with my care pathway, the physio … Today, as a commuter worker, it’s impossible to me to carry out other activities because I arrive so late in the evening that for me the day is already over ... [P01: man, 46 years, EDSS 6.5, North]
- **Improving the social security net**
  - In my opinion, in Italy, we are really in a bad situation as we lack the sense of “welfare". Last summer I went to England, and going around the city was not a problem: there is the disabled service you call and, if you book early, take you where you want in the city. You need, let’s say the shopping at home? You call them, they bring the shopping to you. Hmm ... I wanted to go to London alone by train. I said, "Ok, now you've done 30, you do 31.” This is what we really miss: services at a social level that make everything easy. [P06: woman, 39 years, EDSS 7, North]
- **Facilitate bureaucracy**
  - All this bureaucracy is killing us. [P04: man, 44 years, EDSS 6.0, North]
- **Supporting patient’s autonomy/accessibility**
  - This is what I’m missing. Resources that on a social level that make everything simpler. And I’m not reasoning about big issues, but just concrete, small issues that make a difference. If I should say "I go to Milan alone, from my house", I can’t do it, because I should find a bus that takes me to the railway station, and the buses are not accessible; and then I should… [P06: woman, 39 years, EDSS 7.0, North]

Both

Organization and management

- **Rehabilitation: More physiotherapy/occupational therapy**
  - From a practical point of view, some physio would help me, for sure. [P05: woman, 44 years, EDSS 6.0, North]
  - I always wonder why a center like XX does not have a service of physiotherapy. [P06: woman, 39 years, EDSS 7.0, North]

Empowerment training

- **HPs**
  - Communication (P, N)
    - Surely knowing it, from an authoritative external source, would have given me a little more comfort, by being aware of the period I was going to live. Yes. [P01: man, 46 years, EDSS 6.5, North]
    - I would advise the doctors to undress the gown and to immerge themselves a bit into the daily life of their patients... and therefore to try to be, as well as doctors, friends of their patients. Instead in the relationship doctor-patient, one is limited to describe the state of mind ... no ... not even the state of mind ... the physical state that a person has, labeling the person according to the stage of the disease ... [P01: man, 46 years, EDSS 6.5, North]
    - Maybe it's stupid what I'm about to say, but leave the person in his ignorance. Without insisting on giving information. [P05: woman, 44 years, EDSS 6.0, North]
